# SAMTI: Sampling Adaptive Thermodynamic Integration for Alchemical Free Energy Calculations

**DOI:** 10.1021/acs.jpcb.5c05358

**Published:** 2025-12-09

**Authors:** Tai-Sung Lee, Omid Jahanmahin, Saikat Pal, Darrin M. York

**Affiliations:** Laboratory for Biomolecular Simulation Research, Center for Integrative Proteomics Research, Institute for Quantitative Biomedicine (IQB), and Department of Chemistry and Chemical Biology, Rutgers University, Piscataway, New Jersey 08854, United States

## Abstract

Accurate and efficient calculation of alchemical free energies is a critical challenge in computational chemistry, frequently hindered by the inherent limitations of conventional thermodynamic integration (TI) methods. These limitations include poor phase-space overlap between discrete alchemical states, inefficient allocation of computational resources, and a fundamental time scale separation between alchemical transformations and molecular conformational sampling, which collectively lead to slow convergence and high statistical uncertainty. This work presents sampling adaptive thermodynamic integration (SAMTI), a unified computational framework designed to systematically overcome these challenges. SAMTI synergistically integrates four components: (1) serial tempering (ST) with a fine-grained alchemical grid to ensure phase-space continuity; (2) variance adaptive resampling (VAR) to dynamically allocate computational effort to high-uncertainty regions; (3) replica exchange (RE) to enhance conformational sampling; and (4) alchemical enhanced sampling (ACES) to resolve kinetic bottlenecks by selectively scaling torsional energy barriers. We evaluated SAMTI’s performance against conventional TI across a benchmark suite of eight molecular systems of increasing complexity, including ion solvation, small molecule annihilation, and challenging protein–ligand transformations. The results demonstrate that SAMTI variants reduce statistical error by 40–75% and, for the most complex systems, the complete ST+VAR+RE (mACES) configuration consistently achieves chemical accuracy (σ_Δ*G*_ < 0.1 kcal/mol) within 10 ns of the total simulation time, a challenging task for conventional methods. Despite using a finer alchemical discretization, SAMTI achieves superior computational efficiency through adaptive resource allocation and faster convergence while automating the optimization of the alchemical pathway. By providing a robust, automated, and reliable solution to both alchemical and conformational sampling challenges, SAMTI establishes a new benchmark for free energy calculations, positioning it as a powerful tool for accelerating molecular design in drug discovery and materials science.

## Graphical abstract

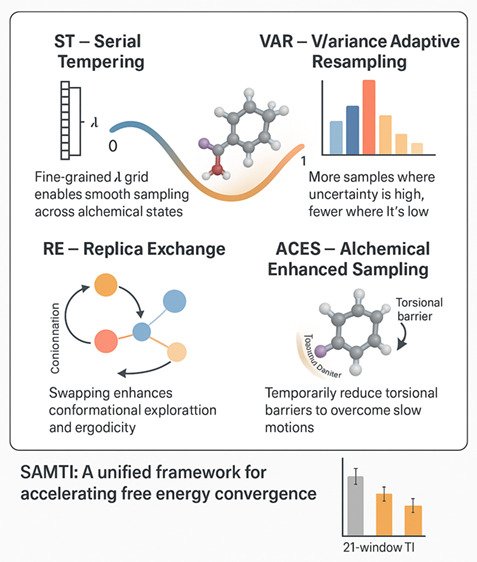

## 1 Introduction

Molecular dynamics (MD) simulations have been pivotal in computational chemistry since their initial demonstration,^1^ offering atomistic-level insights into chemical and biological processes.^2–6^ Among the applications that entail significant computational challenges are free energy calculations,^7–10^ which are utilized in drug design, catalyst development, and the characterization of thermodynamic properties.^11–16^ Recent perspectives and best-practice reviews provide comprehensive guidance for modern alchemical free energy applications in drug discovery, including methodological overviews, software advances, and community recommendations.^16–19^

Thermodynamic Integration (TI) is a rigorously exact method for calculating free energy differences between chemical states.^20^ Although Kirkwood established the theoretical foundation, subsequent methodological advancements, such as the development of soft core potentials to address end point singularities, have expanded its applicability. Nonetheless, TI continues to pose practical challenges.^21–24^ The method is based on the following relationship:

$$\Delta G=\int_{0}^{1}{\left\langle \frac{\partial U(\lambda )}{\partial \lambda }\right\rangle }_{\lambda }d\lambda$$ 1

where *U*(λ) represents the potential energy as a function of the alchemical coupling parameter λ, and ⟨·⟩_λ_ denotes the ensemble average for a given λ. Despite its strong theoretical basis, TI encounters practical issues that impact its accuracy and computational efficiency, particularly in relation to phase space sampling, phase overlapping, and convergence of the calculated free energy.

### 1.1 Fundamental Challenges in Free Energy Calculations

#### 1.1.1 Sampling Limitations in Molecular Simulations

Molecular dynamics (MD) simulations are proficient in modeling time-dependent behaviors; however, they face challenges in sampling infrequently occurring conformational states that are separated by substantial activation energies.^10,25,26^ This limitation is particularly pronounced in free energy calculations, where the incomplete sampling of high-energy states leads to slow convergence and estimates with high variance, especially in the context of complex molecular transformations.^27–29^

Monte Carlo (MC) methods can complement MD by sampling equilibrium properties through moves that do not necessarily adhere to physical pathways. However, their efficiency may be compromised in dense systems due to high rejection rates of proposed moves.^26,30^ Temporal correlations in MD trajectories further complicate sampling, necessitating a balance between simulating dynamic processes and achieving statistical convergence.^25^ In alchemical free energy calculations, which involve simulating nonphysical intermediate states along the λ coordinate, sufficient sampling in each λ window is essential.^26,31^

Several specialized techniques, such as replica exchange molecular dynamics (REMD),^32–34^ metadynamics,^35^ and adaptive biasing force (ABF), address these limitations by enhancing sampling over activation energies.^31,36^ Hybrid MD-MC methods integrate features of both simulation types to improve sampling efficiency while maintaining the ability to generate dynamic information.^26,37,38^

#### 1.1.2 Bottlenecks in Alchemical Free Energy

Window-based alchemical free energy simulations, such as thermodynamic integration (TI) calculations, are highly sensitive to the choice of λ discretization schemes (λ-spacing), which can present challenges for accurate free energy estimation. Insufficient phase space overlap between adjacent λ windows may result in sampling discontinuities, potentially introducing systematic errors in free energy calculations and increasing the variance of the derivative $\frac{\partial U}{\partial \lambda }$ , thereby adversely affecting convergence rates.^27,39,40^ This issue stems from inadequate exploration of transitional states between the initial and final thermodynamic states, which is particularly pertinent to complex biomolecular systems. Conversely, an excessively fine discretization of λ can lead to substantial computational costs without corresponding improvements in accuracy. The difficulty is exacerbated for nonlinear energy landscapes, such as those encountered during particle creation or annihilation or with the use of soft core potentials, where the energy barriers may necessitate a higher density of λ points in specific regions. Identifying these high variance regions prior to simulation is often challenging, potentially requiring computationally intensive iterative optimization procedures that increase overall computational cost.^24,41^

Moreover, the optimal λ distribution is highly system-dependent, varying considerably, for instance, between solvation systems and protein–ligand binding systems. This variability is also influenced by the selected alchemical pathway, such as linear coupling, soft core potentials, or other transformation protocols, which affect the free energy landscape. Consequently, TI protocols optimized for specific systems often exhibit limited transferability and may necessitate substantial recalibration of new molecular systems. This lack of generalizability can be a limitation in high-throughput drug discovery applications where standardized protocols are frequently employed.^42^ The combined challenges of optimal spacing determination, variance reduction, and system-specific optimization underscore the need for TI frameworks with enhanced adaptability that can address these issues with reduced manual intervention.

### 1.2 Established Strategies for Addressing Challenges in Alchemical Free Energy Simulations

#### 1.2.1 Enhanced Sampling Techniques

Specialized sampling strategies have been developed to address convergence challenges in thermodynamic integration (TI). One such strategy is Hamiltonian replica exchange (HRE), which enhances configurational sampling by simultaneously executing multiple molecular dynamics simulations at distinct alchemical coupling parameter (λ) values. Exchange moves between adjacent replicas are performed using Monte Carlo criteria to promote overlap in phase space.^33,43,44^ This mechanism facilitates the movement of molecular configurations across the λ range, enabling the system to overcome kinetic barriers and improve the convergence properties. However, HRE typically incurs a computational cost that scales linearly with the number of replicas, presenting limitations for large systems or high-throughput workflows.

An alternative approach is serial tempering, wherein a single replica transitions between λ states based on predefined statistical weights. This method can reduce computational resource demands while retaining some of the enhanced sampling benefits associated with replica exchange.^45,46^ Nonetheless, determining optimal weighting schemes remains a key challenge, often requiring manual tuning to ensure uniform state visitation. The absence of automated robust schemes for weight adaptation has limited the widespread application of serial tempering despite its potential advantages.

#### 1.2.2 Optimal Usage of Computational Resources

A significant advancement in contemporary free energy calculations is the dynamic optimization of computational resources through automated, data-driven workflows. These methodologies aim to mitigate the substantial costs associated with fixed-length simulations by determining optimal stopping points in real-time. For example, the convergence-adaptive roundtrip (CAR) method developed by Yao et al. employs ongoing convergence analysis to automatically adjust simulation durations, thereby facilitating the rapid propagation of conformations and reportedly achieving an over 8-fold increase in the speed of FEP calculations.^47^ Similarly, Koby et al. have introduced an iterative thermodynamic integration workflow that utilizes automatic equilibration detection and convergence testing with statistical metrics such as the Jensen-Shannon distance. This approach allows each alchemical window’s simulation to conclude once a predefined precision is attained, a strategy demonstrated to reduce computational costs by over 85% while maintaining accuracy.^48^ Collectively, these adaptive strategies represent a pivotal shift toward intelligent resource allocation, enabling a more targeted and efficient use of computational power, which is essential for high-throughput drug discovery applications.

#### 1.2.3 λ-Dynamics and Continuous Alchemical Coordinate Sampling

A distinct alternative to discrete λ-window methods is λ-dynamics, in which the alchemical coupling parameter λ is treated as a continuous dynamical variable that evolves alongside the system’s coordinates.^49^ This formulation allows for direct sampling over the entire alchemical space within a single trajectory, eliminating the need for predefined λ windows. Multistate λ-dynamics (MSLD) further extends this concept by allowing multiple ligand transformations to be sampled concurrently, thereby increasing computational efficiency in relative binding free energy calculations.^50,51^ In these implementations, each ligand is associated with a distinct λ variable, and transitions between chemical states occur dynamically.

While λ-dynamics avoids the sampling discontinuities associated with window-based methods, it introduces new requirements related to the design of the λ potential energy surface. Inadequate exploration of the λ dimension can lead to sampling inefficiencies, necessitating the use of biasing techniques such as adaptive biasing force (ABF) or metadynamics to improve coverage.^31^ Recent hybrid approaches like Lambda-ABF-OPES have demonstrated significant improvements, achieving up to 9-fold enhancement in sampling efficiency by combining adaptive biasing with on-the-fly probability enhanced sampling.^52^ Furthermore, well-tempered metadynamics combined with λ-ABF (WTM-λABF) has shown the capability to handle transformations with up to 1000 intermediates efficiently.^53^ When appropriately parametrized, λ-dynamics offers a flexible framework for continuous-state alchemical simulations, particularly when integrated with adaptive or automated sampling enhancements.

#### 1.2.4 Advances in λ-Dynamics and Adaptive Biasing

Recent methodological advancements have aimed to enhance both the statistical efficiency and computational feasibility of λ-dynamics-based free energy estimation. Ding et al. introduced a Gibbs sampler-based λ-dynamics (GSLD) framework, wherein λ can be modeled as either a continuous or discrete variable.^54^ In GSLD, the joint distribution of atomic coordinates and λ is sampled through alternating updates of coordinates and λ. A significant contribution of this work was the Rao-Blackwell estimator (RBE), which estimates free energies based on the trajectory of atomic coordinates rather than λ transitions, resulting in variance reduction in certain instances. Furthermore, the authors demonstrated that the multistate Bennett acceptance ratio (MBAR) and unbinned weighted histogram analysis method (UWHAM) equations can be derived as special cases of RBE. For continuous λ variants, the method facilitates the simultaneous evaluation of multiple ligand transformations by using automatically generated biasing potentials derived via a Wang–Landau-type algorithm.

To address the high free energy barriers encountered in multisite alchemical simulations, Hayes et al. developed an adaptive landscape flattening (ALF) method.^55^ This technique introduces system-specific biasing potentials, including fixed, quadratic, and end point trap terms, to mitigate barriers associated with significant structural perturbations, such as those involving changes in ligand volume or flexibility. The bias coefficients are iteratively optimized based on sampling feedback. The approach also incorporates solutions to common error sources, including end point trapping (addressed by sharp bias terms) and solvent-related artifacts from hard-core potentials (resolved via a novel soft-core potential that applies λ-dependent remapping within a restricted distance range).

More recently, Robo et al. introduced a dynamic biasing extension of GSLD, termed LaDyBUGS (bias-updated Gibbs sampling λ-dynamics).^56^ This method continuously updates biasing potentials during the simulation, eliminating the need for separate presimulation bias estimation. The sampling protocol alternates between molecular dynamics of atomic coordinates at fixed λ and resampling of λ based on potential energies of all available alchemical states. Following each λ resampling step, bias potentials are updated by using free energy estimates derived from FastMBAR, with initial flat biases progressively refined as the simulation advances. The LaDyBUGS algorithm is implemented in OpenMM and enables efficient sampling of multiple ligand states by leveraging a strongly connected graph representation of transformation pathways.

#### 1.2.5 Advances in λ Protocol Optimization

Recent methodological advancements have focused on dynamic optimization of λ spacing and the allocation of computational resources during simulations. Adaptive Lambda Scheduling (ALS) exemplifies this approach by adjusting λ distributions based on ongoing assessments of the free energy landscape, thereby enhancing efficiency in relative binding free energy (RBFE) calculations.^57^ Similarly, the automated adaptive λ method for relative free energy perturbation (RFEP) developed by Zeng et al. employs initial short simulations to identify regions of interest, followed by a split and merge algorithm that allocates more sampling to high-variance λ windows and less to converged regions.^58^ Complementary approaches include methods for optimizing alchemical intermediate spacing based on thermodynamic length principles.^59^ Additionally, Zhang et al. extended the alchemical enhanced sampling (ACES) method,^61^ by integrating it with optimized phase space overlap (Opt PSO) criteria, designing λ spacing to maximize exchange acceptance rates between adjacent states.^61^ Concurrently, the λ adaptive biasing force (λ-ABF) framework by Lagardère et al. combines adaptive biasing with λ dynamics, offering a method that dynamically applies biasing forces along the alchemical coordinate to accelerate convergence.^31^ Collectively, these methodologies signify a shift toward more autonomous λ optimization, reducing the necessity for manual intervention and enhancing computational efficiency across various molecular systems.

### 1.3 Summary of Current Limitations

Despite decades of development, conventional thermodynamic integration methods are hindered by three fundamental limitations that significantly affect accuracy and efficiency: (1) **Phase space overlap problems**: Sparse λ discretization (typically 10–30 windows) results in inadequate overlap between adjacent states, leading to sampling discontinuities and systematic errors, while dense discretization becomes computationally prohibitive; (2) **Inefficient resource allocation**: Uniform sampling allocation results in wasted computational effort in converged regions while undersampling high-variance regions where accuracy is most critical; and (3) **Conformational sampling bottlenecks**: Slow torsional motions and kinetic barriers create convergence failures that cannot be resolved by alchemical sampling improvements alone.

These limitations become increasingly pronounced for complex biomolecular transformations, where conventional methods often necessitate impractically long simulations to achieve chemical accuracy (σ_Δ*G*_ < 0.1 kcal/mol), often incurring significant supercomputer time and still failing to converge. Existing enhancement strategies typically address only one limitation at a time, failing to capture the synergistic benefits possible from integrated solutions.

### 1.4 Proposed Solution: SAMTI

To systematically address the persistent challenges in thermodynamic integration, we introduce the SAMTI approach (Figure 1). SAMTI is an integrated computational framework that systematically addresses the three primary limitations of conventional TI: (1) **inadequate phase-space overlap** between adjacent λ windows, addressed by the ST (serial tempering) component using fine-grained λ grids; (2) **inefficient resource allocation**, addressed by the VAR (Variance Adaptive Resampling) component that dynamically prioritizes high-uncertainty regions; and (3) **poor conformational sampling**, addressed by the RE (Replica Exchange) component that enhances exploration of complex energy landscapes. By adapting to system-specific free energy landscapes and variance distributions, SAMTI is designed to achieve improved convergence rates while maintaining accuracy standards relevant for computational chemistry and drug discovery applications. Figure 1 provides a schematic overview of the SAMTI framework’s four integrated components and their workflow, illustrating how initialization, adaptive sampling (ST+VAR), replica exchange (RE), and alchemical enhanced sampling (ACES) work together to achieve robust free energy calculations.

**Figure 1:**
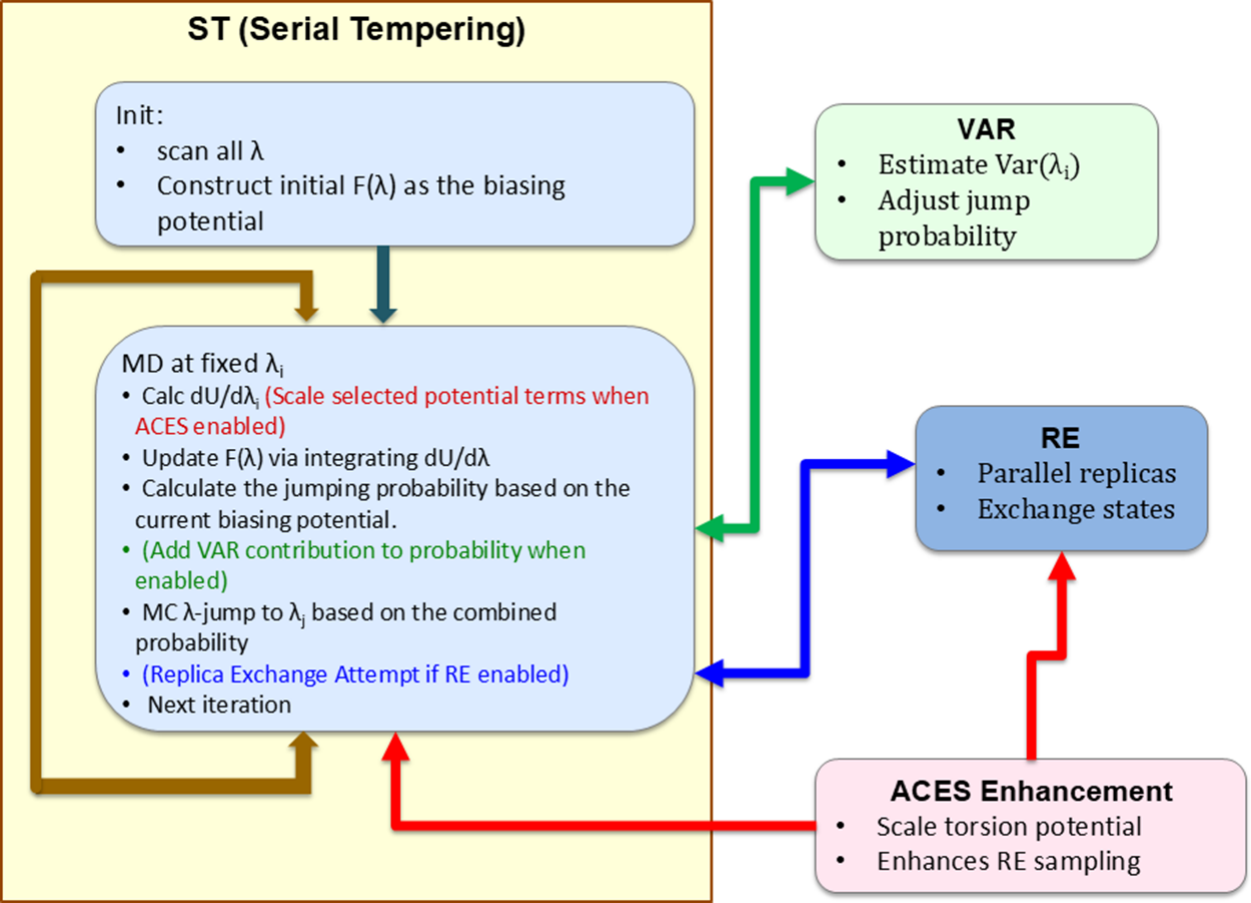
SAMTI workflow with integrated components. **ST (Serial Tempering)** orchestrates the core loop (left panel): *Init* scans all λ and constructs an initial bias *F*(λ); at fixed λ_*i*_ the simulation (i) computes ⟨∂*U*/∂λ⟩_*i*_, (ii) updates *F*(λ) by integrating ∂*U*/∂λ, and (iii) evaluates MC jump probabilities from the current bias, performs an MC jump λ_*i*_ → λ_*j*_, and iterates. **VAR** (green) estimates Var­(λ_*i*_) and adds a variance-weighted contribution to the jump probabilities when enabled (adaptive resource allocation). **RE** (blue) runs parallel replicas and attempts to exchange full states between replicas at set intervals when enabled. **ACES** (red) scales selected torsional terms (Hamiltonian modification; γ-scaling) to lower conformational barriers, strengthening both the ST loop and RE efficiency when enabled. Colored arrows match the component contributions (green = VAR, blue = RE, red = ACES); ST remains the primary driver, while the other modules are optional.

#### 1.4.1 ST (Serial Tempering)

The Serial Tempering (ST) component is conceptually inspired by prior developments in enhanced sampling methodologies, including serial tempering,^45,46^ adaptive biasing techniques,^62^ and bias-updated λ-dynamics frameworks such as GSLD^54^ and LaDyBUGS.^56^ The ST protocol employs a finely discretized λ grid—typically comprising 100–200 windows—to enhance phase space overlap along the alchemical pathway. Sampling is performed via a serial tempering approach,^63^ which alternates between molecular dynamics at fixed λ values and Monte Carlo transitions in λ space.

The ST algorithm comprises four stages: (1) an initial scan to estimate an empirical free energy profile over the discretized λ space; (2) calculation of exchange probabilities between adjacent λ states using instantaneous potential energies, guided by the current free energy estimate as a biasing potential; (3) refinement of the free energy profile based on empirical visitation statistics; and (4) dynamic updating of transition weights to achieve approximately uniform sampling across all λ windows. This iterative scheme is intended to adaptively optimize sampling efficiency during the course of a single simulation, thereby reducing the dependence on manual tuning of λ spacings and facilitating a thorough exploration of both configurational and alchemical spaces.

#### 1.4.2 VAR (Variance Adaptive Resampling)

The VAR component implements a variance-responsive procedure that dynamically allocates computational resources based on the uncertainty in $\frac{\partial U}{\partial \lambda }$ measurements. Grounded in the statistical principle of variance-weighted sampling, also known as optimal allocation or Neyman allocation,^64–67^ VAR constructs an adaptive biasing potential where sampling probabilities are inversely weighted by the local variance of the energy derivative. This approach allocates more sampling effort to regions of high uncertainty, where $\frac{\partial U}{\partial \lambda }$ exhibits larger fluctuations and less sampling in low-variance regions that are closer to convergence. Variance estimates are updated during the simulations, forming a feedback mechanism for the progressive optimization of resource allocation. When combined with ST, this ST+VAR composite is designed to promote uniform λ space coverage and reduce the aggregate uncertainty in free energy estimates, aiming for improved convergence relative to fixed-weight sampling approaches.

#### 1.4.3 RE (Replica Exchange Enhancement)

The RE component enhances parallel efficiency by concurrently executing multiple independent ST or ST+VAR simulations with periodic attempts at replica exchange based on a generalized ensemble framework. This parallel architecture provides two main advantages: (1) replicas periodically exchange conformational states using a Hamiltonian-based Metropolis criterion, which can allow conformations to overcome local energy minima by transitioning to different λ environments; and (2) the independent sampling trajectories can collectively explore a broader region of phase space compared to single replica approaches. The exchange mechanism is designed for low communication overhead to maintain computational efficiency while improving conformational sampling. RE is designed for scalability on high-performance computing resources, enabling SAMTI to be applied to more complex biomolecular systems by increasing the number of replicas without requiring algorithmic modifications. This combination of improved sampling and parallel efficiency makes RE suitable for systems characterized by complex energy landscapes or slow conformational transitions.

#### 1.4.4 ACES (Alchemical Enhanced Sampling)

A primary challenge identified in complex ligand transformations is the temporal disparity between the alchemical and conformational sampling. Although enhanced sampling along the λ coordinate can mitigate issues related to variance and phase-space overlap, conformational barriers with time scales surpassing the duration of simulations necessitate further enhancement. The ACES (alchemical enhanced sampling) component, derived from the methodology of Lee et al.,^60^ addresses this challenge by selectively scaling torsional potential energy terms to generate enhanced sampling states that facilitate conformational transitions otherwise kinetically hindered within simulation time scales.^61,68^ ACES can be applied to target individual critical torsions (sACES) or multiple cooperative torsional coordinates (mACES), contingent upon the complexity of the conformational change requisite for alchemical transformation.

We propose that the synergistic integration of these four components will yield significant advancements over traditional thermodynamic integration methods: (1) **Statistical accuracy**: The integration of fine-grained λ-spacing (ST), variance-proportional resource allocation (VAR), enhanced conformational sampling (RE), and conformational barrier reduction (ACES) will result in a marked reduction in statistical errors compared to conventional 21-window TI methods; (2) **Computational efficiency**: Despite utilizing 5× more λ windows, adaptive resource allocation and accelerated convergence will sustain comparable or enhanced computational efficiency per unit accuracy; and (3) **Systematic performance scaling**: Improvements will scale with molecular complexity, with the comprehensive ST+VAR+RE (mACES) configuration offering the most substantial benefits for challenging transformations involving conformational barriers.

This paper delineates SAMTI’s complete theoretical framework (Section 2), implementation specifics (Section 3), and performance evaluation across eight molecular systems, ranging from simple ion solvation to complex protein–ligand transformations with enhanced sampling protocols (Section 4). We demonstrate that the synergistic combination of all four components (ST, VAR, RE, and ACES) within the complete ST+VAR+RE (mACES) configuration achieves a significant reduction in statistical errors compared with conventional 21-window TI methods, with the complete framework consistently attaining high accuracy for complex transformations. Section 5 discusses the relative contributions of each component and establishes ST+VAR+RE with ACES as a comprehensive solution for addressing both alchemical and conformational sampling challenges.

To elucidate how SAMTI addresses these multidimensional sampling challenges at a fundamental level, the following section establishes the mathematical foundations underlying SAMTI’s four components and their integration. We commence with the statistical mechanics basis of thermodynamic integration, develop the theoretical framework for each adaptive component, and conclude with an algorithmic implementation framework that bridges theory and practice.

## 2 Theory

### 2.1 Statistical Mechanics Foundation of Thermodynamic Integration

The theoretical basis of thermodynamic integration is derived from the work of Kirkwood et al., which relates free energy differences to ensemble averages of Hamiltonian derivatives. For a system described by a parameter-dependent Hamiltonian *H*(**r**, λ) that transforms continuously between states λ = 0 and λ = 1, the Helmholtz free energy difference is given by the integral relationship:

$$\Delta G=G(1)-G(0)=\int_{0}^{1}{\left\langle \frac{\partial H(r,\lambda )}{\partial \lambda }\right\rangle }_{\lambda }d\lambda$$ 2

where ⟨·⟩_λ_ represents the canonical ensemble average evaluated at a fixed λ value.^20^ This formulation converts the free energy calculation into an integration problem along an alchemical pathway, forming the basis for TI methodologies. The ensemble average at each intermediate λ state is defined by the configurational integral:

$${\left\langle \frac{\partial H}{\partial \lambda }\right\rangle }_{\lambda }=\frac{\int \frac{\partial H(r,\lambda )}{\partial \lambda }e^{-\beta H(r,\lambda )}dr}{\int e^{-\beta H(r,\lambda )}dr}$$ 3

where β = (*k*_B_ *T*)^−1^, with *k*_B_ being the Boltzmann constant and *T* the absolute temperature, and **r** denotes the coordinates of the system in configuration space. This derivative ensemble average corresponds to a generalized force along the alchemical coordinate, and its statistical convergence affects the accuracy of the free energy estimates. The Hamiltonian often takes the functional form *H*(**r**, λ) = (1 – λ)*H*_0_(**r**) + λ*H*_1_(**r**) for linear interpolation between end points. Soft core potentials are frequently used to prevent singularities, for instance, during particle creation or annihilation processes.^22,24,41^ The theoretical validity of TI depends on the continuous differentiability of the Hamiltonian with respect to λ and ergodic sampling at all intermediate states. These conditions can be difficult to satisfy in complex biomolecular systems with complex energy landscapes.

### 2.2 SAMTI Theoretical Framework

#### 2.2.1 ST Component Theory

The Serial Tempering (ST) component is an implementation of Gibbs sampling, adapted from temperature-based serial tempering^45^ to operate along the alchemical coordinate λ. ST alternates between two modes: (1) molecular dynamics propagation at fixed λ values for configurational exploration, and (2) Monte Carlo style λ jumps subject to adaptive biasing potentials. This dual sampling strategy is designed to promote exploration of both the conformational and alchemical dimensions.

The ST algorithm alternates between two distinct phases:

**Phase 1: Sequential Scanning** – The system systematically visits λ windows in order (λ_1_ → λ_2_ →··· → λ_*N*_ → λ_1_) to build initial bias estimates and establish basic connectivity.

**Phase 2: Biased Monte Carlo Jumps** – After sufficient scanning, the algorithm switches to Monte Carlo λ jumps using accumulated bias potentials. The normalized (“heat-bath”) jump probability from current state λ_*i*_ to candidate state λ_*j*_ is calculated as

$$P_{\mathrm{jump}}\left(\lambda_{i}\to \lambda_{j}\right)=\frac{\exp\left(\mathrm{logP}\left(\lambda_{j}\right)\right)}{\underset{k\neq i}{\sum }\exp\left(\mathrm{logP}\left(\lambda_{k}\right)\right)}$$ 4

where logP­(λ_*j*_) = −β­[*U*(**r**, λ_*j*_) – *U*(**r**, λ_*i*_) + *F*_*j*_ – *F*_*i*_] represents the log-probability including both energetic and bias contributions. (An equivalent pairwise Metropolis acceptance using the same energy-plus-bias difference yields the same stationary distribution; the normalized form is used here for convenience and efficient multitarget proposals.)

#### 2.2.2 Biasing Potential Construction

The biasing potential in ST is constructed as the negative of the free energy function obtained by integrating the thermodynamic derivative along the alchemical coordinate:

$$F_{i}=-\int_{0}^{\lambda_{i}}{\left\langle \frac{\partial U}{\partial \lambda }\right\rangle }_{\lambda }d\lambda$$ 5

This biasing potential effectively flattens the free energy landscape, enabling uniform sampling across all of the λ windows. The integration is performed using Simpson’s rule for numerical accuracy:

$$F_{i}=F_{i-2}-\frac{\Delta \lambda }{3}\left[{\left\langle \frac{\partial U}{\partial \lambda }\right\rangle }_{i-2}+4{\left\langle \frac{\partial U}{\partial \lambda }\right\rangle }_{i-1}+{\left\langle \frac{\partial U}{\partial \lambda }\right\rangle }_{i}\right]$$ 6

where Δλ = 0.01 is the spacing between adjacent windows (as described in Methods Section 3.2). The biasing potential compensates for the intrinsic free energy differences between λ states, allowing the system to explore all regions of alchemical space with equal probability. This approach eliminates the need for iterative feedback mechanisms, as the bias is directly derived from the underlying thermodynamics.

#### 2.2.3 Practical Considerations and Relation to Parallel Tempering

Compared to parallel tempering, this serial implementation does not require simultaneous replica simulations, which can reduce overhead while retaining phase-space mixing between thermodynamic states. In the full SAMTI framework, multiple independent ST simulations may still be run in parallel when combined with a replica exchange (RE). The efficiency of λ-space exploration depends on the frequency of jump attempts and the magnitude of λ steps, which together trade off diffusion rate versus acceptance probability.

#### 2.2.4 VAR Component Theory

The variable adaptive response (VAR) component employs a resource allocation strategy that emphasizes sampling in areas characterized by high statistical uncertainty, drawing on optimal allocation principles from sampling theory.

The VAR algorithm persistently evaluates the local variance of the thermodynamic derivative:

$${\mathrm{Var}}_{i}={\left\langle {\left(\partial U/\partial \lambda \right)}^{2}\right\rangle }_{i}-{\left\langle \partial U/\partial \lambda \right\rangle }_{i}^{2}$$ 7

This variance estimate directly quantifies the statistical uncertainty in the integrand and serves as the foundation for resource allocation. The variance estimates are updated dynamically during the simulation by using a running average over the accumulated sampling history at each λ window, allowing the algorithm to adapt to evolving statistical properties as conformational sampling progresses.

#### 2.2.5 Target Probability Calculation

The VAR algorithm determines target probabilities that are directly proportional to the local variance:

$$P_{\mathrm{target}}\left(\lambda_{i}\right)=\frac{{\mathrm{Var}}_{i}}{\underset{j}{\sum }{\mathrm{Var}}_{j}}$$ 8

The modified jumping probability incorporating variance weighting is calculated as

$$P_{\mathrm{jump}}^{\prime }\left(\lambda_{i}\to \lambda_{j}\right)=P_{\mathrm{jump}}\left(\lambda_{i}\to \lambda_{j}\right)\frac{P_{\mathrm{target}}\left(\lambda_{j}\right)}{P_{\mathrm{target}}\left(\lambda_{i}\right)}$$ 9

where *P*_jump_(λ_*i*_ → λ_*j*_) is the base ST jump probability from eq 3. The practical implementation of this variance-weighted probability adjustment, including the minimum probability constraint, is detailed in Methods Section 3.2. This direct proportionality to variance facilitates optimal resource allocation for minimizing integration variance under the premise that sampling effort should be concentrated where statistical uncertainty is greatest.

#### 2.2.6 Theoretical Foundation for Variance-Based Optimization

The effectiveness of the VAR can be understood through error propagation theory. For a discretized thermodynamic integration with *N*_λ_ windows, the total variance of Δ*G* is

$$\sigma_{\Delta G}^{2}=\overset{N_{\lambda }}{\underset{i=1}{\sum }}\left(\frac{\Delta \lambda_{i}}{N_{i}}\right)\sigma_{i}^{2}$$ 10

where Δλ_*i*_ is the λ interval for window *i*, *N*_*i*_ is the number of uncorrelated samples in window *i*, and σ_*i*_^2^ is the variance of ∂*U*/∂λ in window *i*.

The VAR strategy minimizes σ_Δ*G*_^2^ by distributing computational effort proportional to local variance: *N*_*i*_ ∝ σ_*i*_^2^ (for constant Δλ_*i*_). This allocation equalizes the contribution $\frac{\Delta \lambda_{i}\sigma_{i}^{2}}{N_{i}}$ across all windows, ensuring uniform marginal reduction in variance per unit computational effort. This theoretical framework establishes VAR’s advantage over uniform sampling, particularly for systems with heterogeneous variance profiles along the alchemical coordinate.

#### 2.2.7 RE Component Theory

The replica exchange (RE) component enhances SAMTI by introducing a parallel framework wherein multiple independent simulations, or replicas, are executed concurrently with periodic exchanges of configurations. The probability of exchange between configuration **r**_*m*_ at λ_*m*_ and configuration **r**_*n*_ at λ_*n*_ is determined by the Metropolis criterion, which relies solely on the true potential energies:

$$P_{\mathrm{exchange}}=\min\left(1,\exp\left(-\beta \Delta U_{mn}\right)\right)$$ 11

Here, Δ*U*_*mn*_ = *U*(**r**_*n*_, λ_*m*_) + *U*(**r**_*m*_, λ_*n*_) – *U*(**r**_*m*_, λ_*m*_) – *U*(**r**_*n*_, λ_*n*_) represents the potential energy difference for the exchange. Importantly, no biasing potentials are incorporated into the exchange criterion, ensuring that the replica exchange samples from the true thermodynamic ensemble and maintain a detailed balance. This exchange mechanism offers two primary advantages: (1) configurations residing in local minima at one λ value may transition to another λ environment where energy barriers differ, potentially facilitating escape from these minima and (2) conformational states explored by different replicas can be exchanged within the ensemble. Exchange attempts typically occur between adjacent replicas in λ space to sustain higher acceptance probabilities, although alternative exchange schemes can be implemented. RE can function with minimal communication overhead because exchanges are generally attempted infrequently relative to local sampling steps. The replica framework also permits asynchronous adaptation of biasing potentials, wherein each replica updates its bias parameters, and convergence statistics may be shared periodically. This combination of enhanced conformational sampling and parallel execution renders RE suitable for complex biomolecular systems with slow degrees of freedom or kinetic traps that present challenges for single replica approaches.

#### 2.2.8 ACES Component Theory

The ACES component addresses a fundamental limitation in free energy calculations: the sampling of slow conformational degrees of freedom that creates kinetic barriers and conformational traps. ACES operates by creating nonphysical enhanced sampling states where specific potential energy barriers are systematically removed, enabling comprehensive exploration of conformational space that would otherwise be kinetically inaccessible on simulation time scales.

#### 2.2.9 Theoretical Framework

ACES creates enhanced sampling states through the selective scaling of torsional potential energy terms according to

$$V_{\mathrm{torsion}}\left(\gamma \right)=\gamma \times V_{\mathrm{torsion},\mathrm{original}}$$ 12

In this context, γ denotes the enhanced sampling coordinate. At γ = 0, corresponding to dummy states, torsional barriers are entirely removed, facilitating barrier-free rotation. Conversely, at γ = 1, the original torsional potential is completely reinstated. This scaling mechanism permits the system to explore conformational spaces that would otherwise be kinetically inaccessible at a physical state of γ = 1. It is necessary to adjust the scaling of the torsion potentials when employing different λ-scheduling schemes.^41^ In the common direct-mapping schedule, the torsion-scaling coordinate follows the alchemical parameter (γ­(λ) = λ), but more general λ-scheduling mappings γ­(λ) (e.g., nonlinear or piecewise forms) may be used to tailor barrier suppression while TI is still performed along λ.

The ACES methodology can target individual critical torsions (sACES) or multiple cooperative torsional coordinates (mACES), depending on the complexity of the conformational barriers present in the molecular transformation. The choice between sACES and mACES implementations is determined by the number and coupling of the slow conformational degrees of freedom identified in the system.

#### 2.2.10 Hamiltonian Replica Exchange Integration

The HRE framework enables a counterdiffusion of replicas between the real state and the barrier-free dummy state along the γ pathway. This process ensures that the extensive conformational diversity explored in the enhanced-sampling state is effectively transmitted to the physical end states, thereby allowing them to attain a proper Boltzmann-weighted equilibrium distribution.

The replica exchange mechanism follows the standard Metropolis criterion applied to total Hamiltonian differences between the physical and enhanced sampling states. The practical implementation of ACES within the SAMTI framework, including torsion selection criteria and integration with ST+VAR+RE components, is detailed in Methods Section 3.2.

Importantly, free energy calculations integrate solely along the physical alchemical coordinate, as ACES dummy states serve exclusively as enhanced sampling intermediates rather than thermodynamically meaningful states. This ensures that the computed free energies remain physically meaningful while benefiting from an enhanced conformational exploration.

### 2.3 SAMTI Framework Integration

The theoretical underpinning of SAMTI’s efficacy is rooted in the synergistic integration of its four components: ST facilitates adaptive exploration of alchemical space; VAR optimizes resource allocation based on statistical uncertainty; RE enhances conformational sampling through parallelization; and ACES surmounts kinetic barriers in slow conformational degrees of freedom. The practical implementation details of how these components are integrated are delineated in the Methods section.

### 2.4 Algorithmic Implementation Framework

The transition from the theoretical framework to the computational algorithm necessitates careful consideration of numerical implementation, convergence criteria, and parameter selection. This section provides the algorithmic foundation that bridges the theoretical development with the practical implementation described in the Methods section.

#### 2.4.1 SAMTI Master Algorithm

The overall SAMTI algorithm integrates the four components through a hierarchical control structure:

**Algorithm 1: Complete SAMTI Master Algorithm**

1. Initialize λ grid with *N*_λ_ windows (including ACES dummy states if applicable)
2. Perform preliminary scan to estimate initial free energy profile *F*_*i*_^(0)^
3. Initialize variance estimates σ_*i*_^(0)^ from preliminary data
4. For replica *r* = 1 to *N*_rep_:
  - Launch ST+VAR simulation at replica-specific λ distribution
5. While simulation not converged:
  - For each replica in parallel:
    - Perform *N*_cycle_ MD steps at current λ state
    - Attempt λ jump using current biases (ST component)
    - Update local statistics for bias and variance estimation
  - If adaptation interval reached:
    - Update variance estimates σ_*i*_ (VAR component)
    - Update bias potentials *F*_*i*_ using integrated ST+VAR approach
    - Attempt replica exchanges between λ states (RE component)
6. Compute final free energy using thermodynamic integration along physical λ coordinate

Following the establishment of the theoretical framework and algorithmic structure for the comprehensive SAMTI framework, we next elucidate the translation of these concepts into practical implementation. We provide a detailed account of the parameter optimization strategies, initialization protocols, and molecular dynamics setup that ensure robust performance across diverse chemical environments. This implementation section also introduces the eight molecular test systems employed to comprehensively evaluate SAMTI’s capabilities across various transformation types and complexity levels, ranging from simple three-component systems to the complete ST+VAR+RE+mACES framework, addressing both alchemical and conformational sampling challenges.

## 3 Methods

### 3.1 Molecular Systems and Simulation Protocols

Eight molecular systems were selected to evaluate SAMTI’s performance across a range of transformation types and chemical complexities. All molecular dynamics (MD) simulations were performed using a modified version of the AMBER 24 package^69,70^ which implements the SAMTI framework.

#### 3.1.1 Test Systems

The test suite included:

- **Na** ^+^ **Solvation**: An electrostatic decoupling (Na^+^ → dummy) in a 18,034-atom TIP3P water box, serving as a baseline for electrostatic transformations.
- **7CPI Disappearance**: Simultaneous van der Waals and electrostatic decoupling of 7-chloro-1H-indole-2-carboxylic acid phenyl ester in a 14,329-atom TIP4P-EW water box, testing performance on nonbonded interactions with anisotropic solvation.
- **Tyk2 Ligand Transformation (Aqueous and Complex)**: The relative transformation of ejm42 → ejm55 (Figure 2) was studied both in aqueous solution (17,216 atoms, TIP4P-EW) and within the Tyk2 protein binding site. The protein system was prepared from PDB ID 7L0D, with acetyl and *N*-methylamide caps at the N- and C-termini, respectively. The solvated protein system contained 87,584 atoms in a TIP4P-EW water box. These systems assess performance on relative free energy calculations with topological changes in both simple and complex biological environments.
- **ACES Variants**: To evaluate enhanced sampling capabilities, the Tyk2 transformations incorporated alchemical enhanced sampling (ACES). The **sACES** variants targeted a single flexible dihedral angle, while **the mACES** variants targeted two angles. Both were applied in aqueous and protein-bound environments, yielding four additional test cases.

**Figure 2:**
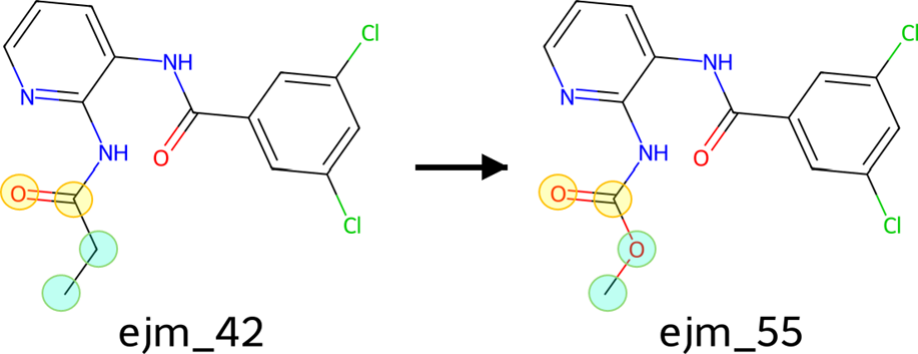
Alchemical transformation of ligand ejm_42 to ejm_55, which is the focus of the ACES simulations. The atoms included in the softcore region for the enhanced sampling protocols are highlighted. The blue-circled atoms are those involved in the standard transformation (i.e., soft core regions for sACES). When ACES is on, the torsion involving these atoms is scaled. The yellow-circled atoms are additional atoms added to the softcore regions for the mACES condition, where an additional torsion is scaled.

#### 3.1.2 System Equilibration Protocol

The following steps were undertaken for the Tyk2-ligand complex system to ensure thorough equilibration: Initially, the energy was minimized using the steepest descent method for 5000 steps, followed by another 5000 steps with the conjugate gradient method to remove any unfavorable contacts. Subsequently, the system was gradually heated from 0 to 300 K in increments of 50 K, with each increment held for 25 ps (150 ps total heating time) while maintaining the canonical ensemble (NVT). During this heating phase, all heavy atoms of the protein–ligand complexes were positionally restrained with a harmonic force constant of 5 kcal mol^–1^ Å^–2^ to their energy-minimized configurations. This was followed by a series of NVT equilibration runs with progressively reduced positional restraint force constants of 2, 1, 0.5, 0.1, and finally, 0 kcal mol^–1^ Å^–2^. Density was then equilibrated through a 1 ns simulation under isothermal–isobaric (NPT) conditions.

For other systems, 2000 energy minimization steps, followed by 1 ns of NVT, and 1 ns of NPT, were performed before production runs.

#### 3.1.3 Simulation Protocol

All simulations employed the NPT ensemble at 298 K and 1 bar. Force field parameters comprised AMBER99SB-ILDN for proteins^71^ and GAFF2 for ligands,^72^ with system-specific water models (TIP3P^73^ or TIP4P-EW^74^). Alchemical transformations employed SSC2 soft-core potentials.^41,75^

All NPT simulations employed Langevin dynamics^76,77^ as a thermostat with a collision frequency of 5 ps^–1^, and the Monte Carlo barostat^78^ with a pressure relaxation time of 2 ps for temperature and pressure control, respectively. When applied, SHAKE^79^ constrained bonds involving hydrogen atoms with a tolerance of 10^–5^ Å. A cutoff radius of 9 Å was used for all short-ranged nonbonded interactions, while long-range electrostatic interactions were treated using the particle mesh Ewald (PME) algorithm.^80,81^ Periodic boundary conditions were enforced in all simulations.

### 3.2 SAMTI Implementation and Parameters

The SAMTI framework utilized 101 equally spaced λ windows (Δλ = 0.01) encompassing the entire alchemical transformation. Initial conformations were generated through 1 ns of equilibration at the λ = 0 and λ = 1 end states, with intermediate states created via linear interpolation of Hamiltonian parameters. A preliminary 20 ps scan at each λ value established the initial free energy profile for adaptive biasing by providing rough estimates of ⟨∂*U*/∂λ⟩ at all λ values, which were integrated to obtain an approximate free energy profile *F*(λ) along the alchemical coordinate. This profile serves as the negative of the initial biasing potential: *V*_bias_ (λ) = – *F*(λ).

#### 3.2.1 Component Configuration

The four SAMTI components were implemented in AMBER as follows:

- **Serial Tempering (ST)**: Implemented via the custom SAMTI flag sams_type = 2 in modified AMBER 24, ST initially conducted a 500,000-step sequential scan across all λ windows to construct the bias potential, then transitioned to biased Monte Carlo jumps attempted every 100 steps (0.2 ps).
- **Variance Adaptive Resampling (VAR)**: Enabled via the custom SAMTI flag sams_variance = 1 in modified AMBER 24, VAR dynamically adjusted the target distribution to be proportional to the local variance of ∂*U*/∂λ, automatically allocating computational resources to regions of highest statistical uncertainty. The implementation follows a two-step process: (1) initial probabilities are calculated as *P*_*i*_ ∝ Var_*i*_ where Var_*i*_ is the variance of ∂*U*/∂λ at window *i*, then (2) a minimum probability constraint is applied as *P*_*i*_ = max­(0.1 × *P*_*i*,max_, *P*_*i*_) where *P*_*i*,max_ is the highest probability among all windows, followed by renormalization to ensure ∑_*i*_ *P*_*i*_ = 1.
- **Replica Exchange (RE)**: In the ST+RE configuration, eight independent ST simulations were run in parallel (replicas). Every 100 steps (0.2 ps), a replica exchange was attempted between adjacent pairs of these independent simulations (1↔2, 3↔4, 5↔6, 7↔8). The exchange involves swapping the entire state (coordinates, velocities, and current λ value) between the two replicas based on a Metropolis criterion. This allows for a more global exploration of the conformational and alchemical space. The “replica-specific λ distribution” refers to the λ probability distribution sampled by each independent ST replica.
- **Alchemical Enhanced Sampling (ACES)**: For Tyk2 systems, ACES (enabled via the standard AMBER 24 flag gti_add_sc = 25) was implemented as Hamiltonian replica exchange between two *states* (physical and dummy) within each replica. Selected torsional potentials were scaled according to *V*_torsion_(γ) = γ × *V*_torsion,original_. At γ = 0 (dummy state), torsional barriers are completely eliminated, enabling barrier-free rotation, while at γ = 1 the original torsional potential is fully restored. Exchanges between the two Hamiltonian states were attempted every 100 steps (0.2 ps). Importantly, the overall number of concurrent replicas remained unchanged across configurations: **SAMTI used 8 replicas** (independent simulations), and **TI used 21 replicas** (windows). This approach follows the methodology developed by Lee et al.^60^ Torsion selection: for the ligand transformation studied here, two torsional angles were chosen based on prior experience and benchmarking, which showed they dominate relevant conformational sampling;^68^ a general protocol for identifying such torsions is beyond the scope of this work.

### 3.3 Benchmarking and Analysis

#### 3.3.1 Reference Methods

SAMTI’s performance was benchmarked against two conventional TI implementations:

- **21W**: Standard TI using 21 equally spaced λ windows.
- **21W+RE**: A 21-window TI enhanced with Hamiltonian replica exchange across all 21 replicas (windows), with exchange attempts every 0.2 ps.

All reference simulations utilized the same molecular dynamics (MD) protocols as the SAMTI runs to ensure direct comparability. While alternative postprocessing methods, such as the Bennett acceptance ratio (BAR)^82^ or the multistate Bennett acceptance ratio (MBAR),^40^ could be applied to these reference trajectories, we anticipate similar conclusions regarding SAMTI’s performance advantages. This is because the fundamental sampling limitations addressed by SAMTI’s adaptive components remain independent of the integration method employed.

Each system and method was simulated for a cumulative duration of 50 ns, with data recorded at intermediate points (2, 3, 6, 10, 20, 30, and 50 ns) to assess convergence. The 50 ns duration represents the total simulation time: for SAMTI-type simulations, it refers to the total simulation time traversing the entire λ axis; for conventional TI-type simulations, it refers to a total of 50 ns of simulation time distributed across the 21 windows (2.38 ns per window). For the analysis of each simulation time length, the initial 10% of the data was discarded uniformly across all SAMTI and conventional TI methods, and the remaining 90% was utilized for analysis. For instance, in the analysis of 50 ns simulations, the first 5 ns of data points were discarded, and the remaining 45 ns were used for analysis.

All simulations were conducted on the nodes of the Amarel cluster at Rutgers, with 4 or 8 Titan and Ampere GPU accelerators on each node. Each simulation condition was run on a single node. For SAMTI simulations, 8 independent simulations were evenly distributed across the available 4 or 8 GPUs on each node. For conventional TI simulations, the 21 λ windows were evenly distributed across the available 4 or 8 GPUs on each node.

#### 3.3.2 Conformational Sampling Diagnostic

To evaluate the thoroughness of conformational sampling, we compared the internal precision of individual simulations with the external reproducibility across independent replicates. For a well-converged set of *n* = 8 simulations, the average standard error calculated from within each run, ⟨SE⟩, should approximate the standard deviation of the mean values calculated across the *n* independent runs, σ_*A̅*_.

The standard error for simulation *i* is calculated as

$${\mathrm{SE}}_{i}=\frac{\sigma_{i}}{\sqrt{N_{\mathrm{eff},i}}}$$ 13

where σ_*i*_ is the standard deviation of ⟨*dU*/*d*λ⟩ within run *i* and *N*_eff,*i*_ is the effective number of independent samples accounting for autocorrelation. The average standard error is then $\langle \mathrm{SE}\rangle =\frac{1}{n}\sum_{i=1}^{n}{\mathrm{SE}}_{i}$ .

The standard deviation of mean values across simulations is

$$\sigma_{\overset{®}{A}}=\sqrt{\frac{1}{n-1}\sum_{i=1}^{n}({\overset{®}{A}}_{i}-\langle \overset{®}{A}\rangle {)}^{2}}$$ 14

where *A̅*_*i*_ is the time-averaged ⟨*dU*/*d*λ⟩ from simulation *i* and $\langle \overset{®}{A}\rangle =\frac{1}{n}\sum_{i=1}^{n}{\overset{®}{A}}_{i}$ .

A significant discrepancy (⟨SE⟩ ≪ σ_*A̅*_) indicates that individual trajectories are confined to metastable states, suggesting incomplete sampling. In this study, the observable of interest was the ensemble average ⟨*dU*/*d*λ⟩ at each λ state. The condition ⟨SE⟩ ≈ σ_*A̅*_ was employed as a necessary criterion for confirming robust conformational sampling.

Having established the theoretical foundations and implementation methodology, the subsequent section presents comprehensive performance results demonstrating how SAMTI’s four adaptive components systematically address the limitations of conventional TI methods.

## 4 Results

### 4.1 Overview

SAMTI exhibits systematic enhancements in convergence properties and statistical accuracy relative to conventional 21-window TI methodologies across all test systems. The performance improvements scale systematically with molecular complexity, delineating distinct performance regimes: **(1) simple systems** (Na^+^ solvation, 7CPI annihilation) demonstrate systematic performance enhancements, with SAMTI variants achieving competitive or superior convergence compared to conventional methods; **(2) aqueous transformations** (42→55_aq_ family) reveal significant performance disparities, with SAMTI achieving convergence where conventional methods do not; and **(3) protein-bound transformations** (42→55_com_ family) exhibit the most substantial benefits, with ST+VAR+RE providing reliable convergence and the complete ST+VAR+RE (mACES) framework delivering optimal performance with fastest convergence rates in the most challenging systems (Table 1).

**Table 1:** Method Abbreviations Are Used in Convergence Analysis Figures

method	description
ST	serial tempering with fine-grained λ spacing (101 windows)
ST+VAR	ST with variance adaptive resampling for optimal computational resource allocation based on ∂*U*/∂λ variance
ST+RE	ST with replica exchange for enhanced conformational sampling
ST+VAR+RE	complete three-component framework combining ST, VAR, and RE
21W	conventional 21-window thermodynamic integration (uniform spacing)
21W+RE	conventional 21W with high-frequency replica exchange

### 4.2 SAMTI Performance across System Complexity

#### 4.2.1 Simple Systems: Establishing SAMTI Foundations

The Na^+^ solvation system (Figure 3) exemplifies the fundamental advantages of SAMTI in a basic molecular context. As indicated in the tabulated results (Figure 3a), SAMTI variants consistently achieve lower statistical uncertainties compared with traditional methods: ST alone results in a final uncertainty of 0.051 kcal/mol, whereas ST+VAR+RE reduces this to 0.031 kcal/mol, which is comparable to the 21W result of 0.031 kcal/mol. The convergence plots (Figure 3b) illustrate a systematically faster approach to equilibrium values, with SAMTI methods demonstrating smooth monotonic convergence, in contrast to the irregular fluctuations observed in conventional TI.

**Figure 3:**
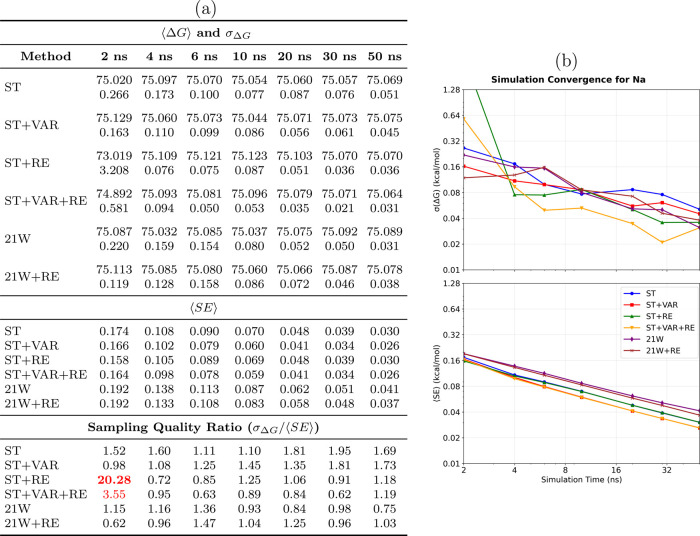
Convergence analysis for the Na^+^ solvation system. **(a) Performance summary**: Averaged free energy differences ⟨Δ*G*⟩ and standard deviations σ_Δ*G*_ (kcal/mol) from 8 independent simulations (upper section); average standard errors ⟨SE⟩ from individual simulations (middle section); sampling quality ratio σ_Δ*G*_/⟨SE⟩ (lower section, red if >2.0, bold red if >5.0). The inequality ⟨SE⟩ ≤ σ_Δ*G*_ serves as a conformational sampling diagnostic: equality indicates all replicates sample identical conformational ensembles, while inequality reveals incomplete exploration. **(b) Temporal convergence**: Upper panel shows σ_Δ*G*_ (inter-replicate variability), lower panel shows ⟨SE⟩ (average within-simulation uncertainty) versus simulation time. **Methods** (Table 1): ST, ST+VAR, ST+RE, ST+VAR+RE, 21W, 21W+RE. **Na** ^+^ **observations**: The decharging process represents an ideal validation case with minimal conformational barriers. SAMTI variants achieve lower uncertainties (ST+VAR+RE: 0.031 kcal/mol) compared with conventional methods (21W: 0.067 kcal/mol) with faster convergence trajectories. Sampling quality ratios (0.75–1.73) indicate excellent conformational sampling across all methods, confirming adequate sampling in this simple electrostatic transformation.

Importantly, the Na^+^ system demonstrates excellent sampling efficiency across all methods, with ⟨SE⟩ ≈ σ_Δ*G*_ relationships observed consistently. This near-equality validates both the robustness of the sampling efficiency diagnostic and confirms the minimal conformational sampling challenges inherent in the Na^+^ decharging process. The absence of significant conformational barriers in this simple electrostatic transformation allows all methods to achieve adequate sampling, providing an ideal baseline for evaluating the statistical framework.

The 7CPI annihilation system (Figure 4) exhibits increased complexity due to heterogeneous solvation environments, which pose challenges to conventional TI methodologies. The performance data (Figure 4a) highlight the pronounced advantages of SAMTI, with ST+VAR achieving an uncertainty of 0.036 kcal/mol compared to 0.067 kcal/mol for 21W at 50 ns, indicating an improvement of approximately 45%. The temporal evolution (Figure 4b) illustrates that conventional methods require more than 40 ns to attain the accuracy that SAMTI variants achieve within 20 ns.

**Figure 4:**
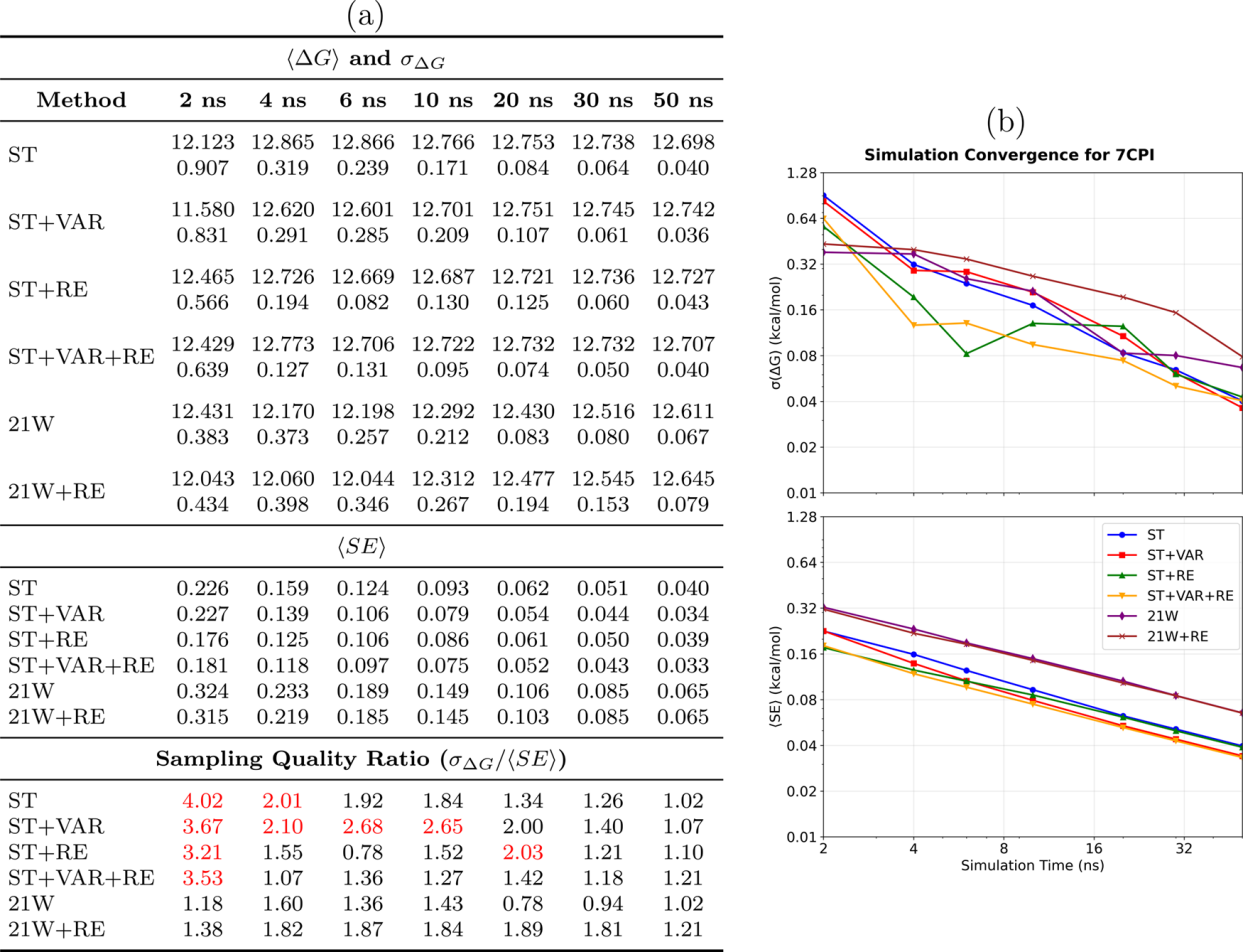
Convergence analysis of the 7CPI annihilation system. **(a) Performance summary**: Averaged ⟨Δ*G*⟩ and σ_Δ*G*_ (kcal/mol) from 8 independent simulations (upper section); ⟨SE⟩ from individual simulations (middle section); sampling quality ratio σ_Δ*G*_/⟨SE⟩ (lower section, red if >2.0, bold red if >5.0). Sampling diagnostics: ⟨SE⟩ ≤ σ_Δ*G*_ (equality = identical conformational sampling). **(b) Temporal convergence**: σ_Δ*G*_ (upper) and ⟨SE⟩ (lower) versus time. **Methods** (Table 1): ST, ST+VAR, ST+RE, ST+VAR+RE, 21W, 21W+RE. **7CPI observations**: The heterogeneous solvation environment creates significant challenges for conventional methods. SAMTI demonstrates pronounced advantages: ST+VAR achieves 0.036 kcal/mol uncertainty versus 0.067 kcal/mol for 21W at 50 ns (45% improvement). Temporal analysis reveals conventional methods require >40 ns to achieve accuracy SAMTI variants reach within 20 ns. Sampling quality ratios (1.02–1.21) indicate excellent conformational sampling across all methods.

Grid resolution does not alter these conclusions: a control calculation employing a denser 201-window layout (7CPI_200) reproduces the 50 ns free energy estimates within the combined statistical uncertainty (see the Supporting Information).

#### 4.2.2 Intermediate Complexity: Aqueous Ligand Transformations

The 42→55_aq_ transformation (Figure 5) represents a notable increase in molecular complexity, involving topological changes that pose significant challenges for sampling. The quantitative results (Figure 5a) indicate substantial performance disparities: while conventional methods exhibit large uncertainties and poor convergence, ST+VAR achieves an impressive precision of 0.013 kcal/mol in 50 ns. The convergence behavior (Figure 5b) reveals the most pronounced performance gaps observed in our test suite, with SAMTI variants converging smoothly, whereas conventional TI methods display persistent oscillations and poor statistical behavior.

**Figure 5:**
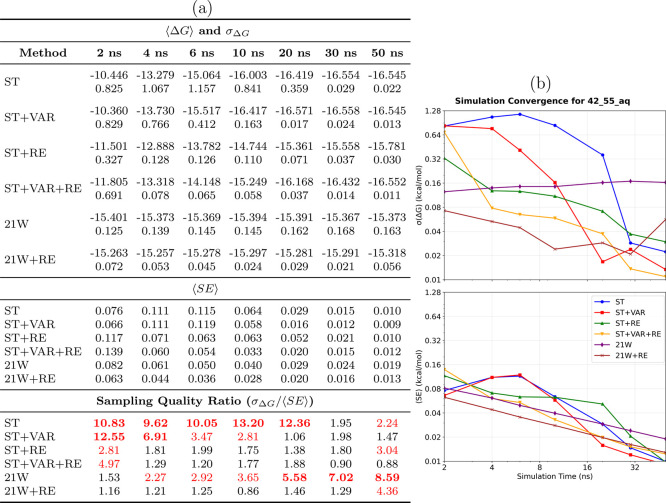
Convergence analysis for the 42→55_aq_ aqueous ligand transformation. **(a) Performance summary**: Averaged ⟨Δ*G*⟩ and σ_Δ*G*_ (kcal/mol) from 8 independent simulations (upper section); ⟨SE⟩ from individual simulations (middle section); sampling quality ratio σ_Δ*G*_/⟨SE⟩ (lower section, red if >2.0, bold red if >5.0). Sampling diagnostic: ⟨SE⟩ ≤ σ_Δ*G*_. **(b) Temporal convergence**: σ_Δ*G*_ (upper) and ⟨SE⟩ (lower) versus time. **Methods** (Table 1): ST, ST+VAR, ST+RE, ST+VAR+RE, 21W, 21W+RE. **42**→**55**_aq_ **observations**: This topologically challenging transformation represents a notable molecular complexity. Performance disparities become dramatic: ST+VAR achieves exceptional precision (0.013 kcal/mol at 50 ns), while conventional methods exhibit large uncertainties with persistent oscillations. **Critical conformational sampling limitation**: Significant deviations between ⟨SE⟩ and σ_Δ*G*_ reveal severe sampling deficiencies without ACES enhancement, with replicas exploring distinct conformational regions rather than comprehensive sampling.

Importantly, this system exposes severe sampling issues in standard SAMTI variants (ST, ST+VAR) and conventional methods (21W) when ACES enhancement is not utilized. Sampling quality ratios exceeding 5.0 (bold red in Figure 5a) reveal that different simulation replicas are exploring distinct conformational regions rather than achieving comprehensive sampling. However, ST+VAR+RE demonstrates acceptable conformational sampling (ratio = 0.92 at 50 ns) despite the conformational complexity, although convergence is slower than with ACES enhancement. These results underscore the fundamental limitation of alchemical methods when faced with slow conformational degrees of freedom and demonstrate that replica exchange partially mitigates sampling deficiencies, even without targeted conformational modifications.

#### 4.2.3 High Complexity: Protein-Bound Ligand Systems

The transition to protein-bound environments significantly increases sampling complexity, as evidenced by the 42→55_com_ system (Figure 6). The tabulated data (Figure 6a) indicate that only advanced SAMTI variants achieve reliable convergence, with ST+VAR+RE reaching an uncertainty of 0.013 kcal/mol, whereas conventional methods exhibit substantially larger statistical errors. The temporal analysis (Figure 6b) demonstrates that the protein binding site introduces additional sampling challenges, which SAMTI components effectively address through enhanced phase space exploration and adaptive resource allocation.

**Figure 6:**
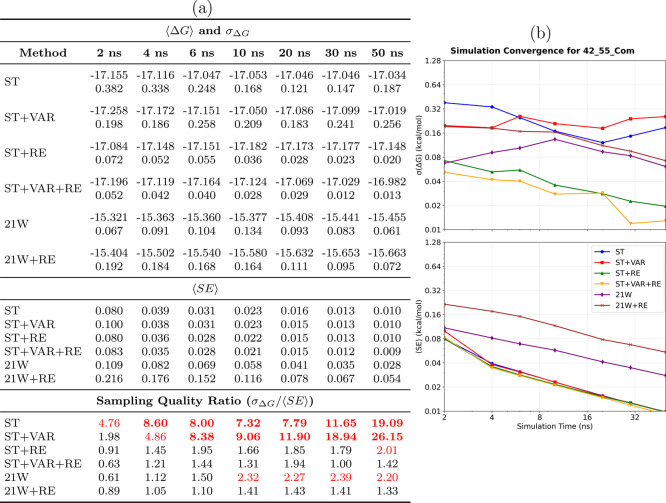
Convergence analysis for the 42→55_com_ protein-bound ligand transformation. **(a) Performance summary**: Averaged ⟨Δ*G*⟩ and σ_Δ*G*_ (kcal/mol) from 8 independent simulations (upper section); ⟨SE⟩ from individual simulations (middle section); sampling quality ratio σ_Δ*G*_/⟨SE⟩ (lower section, red if >2.0, bold red if >5.0). Sampling diagnostic: ⟨SE⟩ ≤ σ_Δ*G*_. **(b) Temporal convergence**: σ_Δ*G*_ (upper) and ⟨SE⟩ (lower) versus time. **Methods** (Table 1): ST, ST+VAR, ST+RE, ST+VAR+RE, 21W, 21W+RE. **42**→**55**_com_ **observations**: Protein binding sites dramatically increase sampling complexity. Only advanced SAMTI variants achieve reliable convergence (ST+VAR+RE: 0.013 kcal/mol uncertainty), while conventional methods exhibit substantially larger errors. SAMTI components effectively address protein-induced challenges through an enhanced phase space exploration. Large deviations between ⟨*SE*⟩ and σ_Δ*G*_ indicate incomplete conformational exploration without ACES, highlighting fundamental challenges of coupled alchemical and conformational sampling in complex biomolecular environments.

Analogous to the aqueous system, the 42→55_com_ transformation without ACES reveals significant sampling deficiencies, with large deviations between ⟨SE⟩ and σ_Δ*G*_ indicating incomplete conformational exploration. The divergent results between SAMTI variants and conventional TI further highlight the fundamental challenges posed by coupled alchemical and conformational sampling in complex biomolecular environments.

### 4.3 Component Analysis: Systematic Performance Improvements

The modular architecture of SAMTI facilitates a systematic assessment of the contributions of the individual components. An analysis encompassing all eight systems indicates that each component confers distinct performance enhancements that correlate with molecular complexity: ST yields a 30–50% improvement over the baseline; VAR contributes an additional 15–25% enhancement in heterogeneous systems; RE achieves 20–40% gains in complex environments; and ACES addresses limitations in conformational sampling beyond the alchemical coordinate.

#### 4.3.1 VAR Component: Adaptive Resource Allocation

The VAR component demonstrates its effectiveness through adaptive sampling density redistribution, as shown in Figure 7. For the Na^+^ system, VAR concentrates sampling in the high-variance middle region (λ ≈ 0.3–0.6), achieving a 4.2× concentration ratio compared to uniform sampling. In the 42→55_aq,sACES_ system, VAR redistributes sampling toward regions of statistical uncertainty, demonstrating how the algorithm automatically detects high-variance λ windows and proportionally allocates computational effort according to Neyman optimal allocation principles (*t*_sampling_(λ) ∝ σ^2^(λ)).

**Figure 7:**
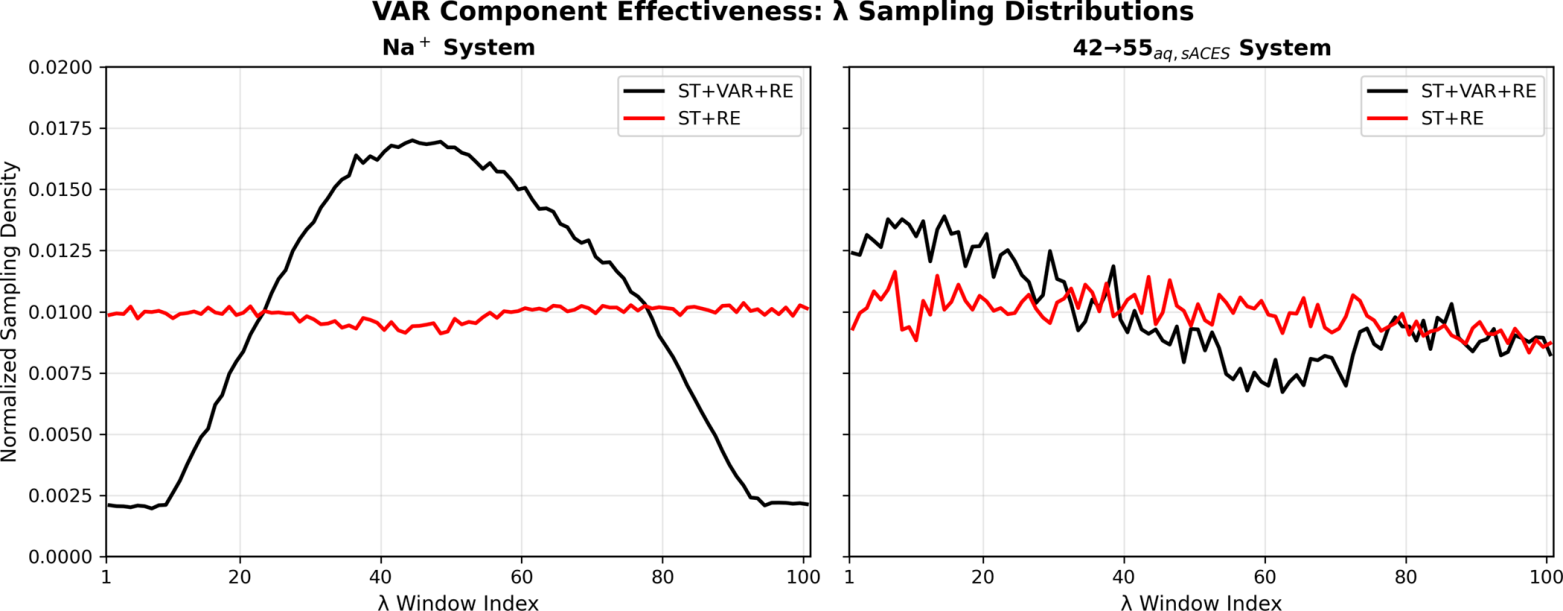
VAR component effectiveness was demonstrated through λ sampling distributions. The figure compares normalized sampling densities across λ windows for methods with (ST+VAR+RE, black solid lines) and without (ST+RE, red solid lines) variance adaptive resampling. Left panel shows the Na^+^ system where VAR concentrates sampling in the high-variance middle region (λ ≈ 0.3–0.6), achieving a 4.2× concentration ratio compared to uniform sampling. Right panel shows the 42→55_aq,sACES_ system where VAR redistributes sampling toward regions of statistical uncertainty, demonstrating adaptive resource allocation. The contrasting patterns illustrate VAR’s mechanism: automatic detection of high-variance regions and proportional allocation of computational effort, implementing Neyman optimal allocation principles.

### 4.4 Addressing Conformational Sampling Limitations

The 42→55 ligand transformation reveals fundamental limitations of both SAMTI and conventional TI methods when confronted with large conformational changes involving slow torsional degrees of freedom. This analysis examines how ACES integration extends SAMTI’s capabilities to address conformational sampling bottlenecks, as demonstrated across the complete family of ACES-enhanced systems (Figures 2 and 8–11).

**Figure 8:**
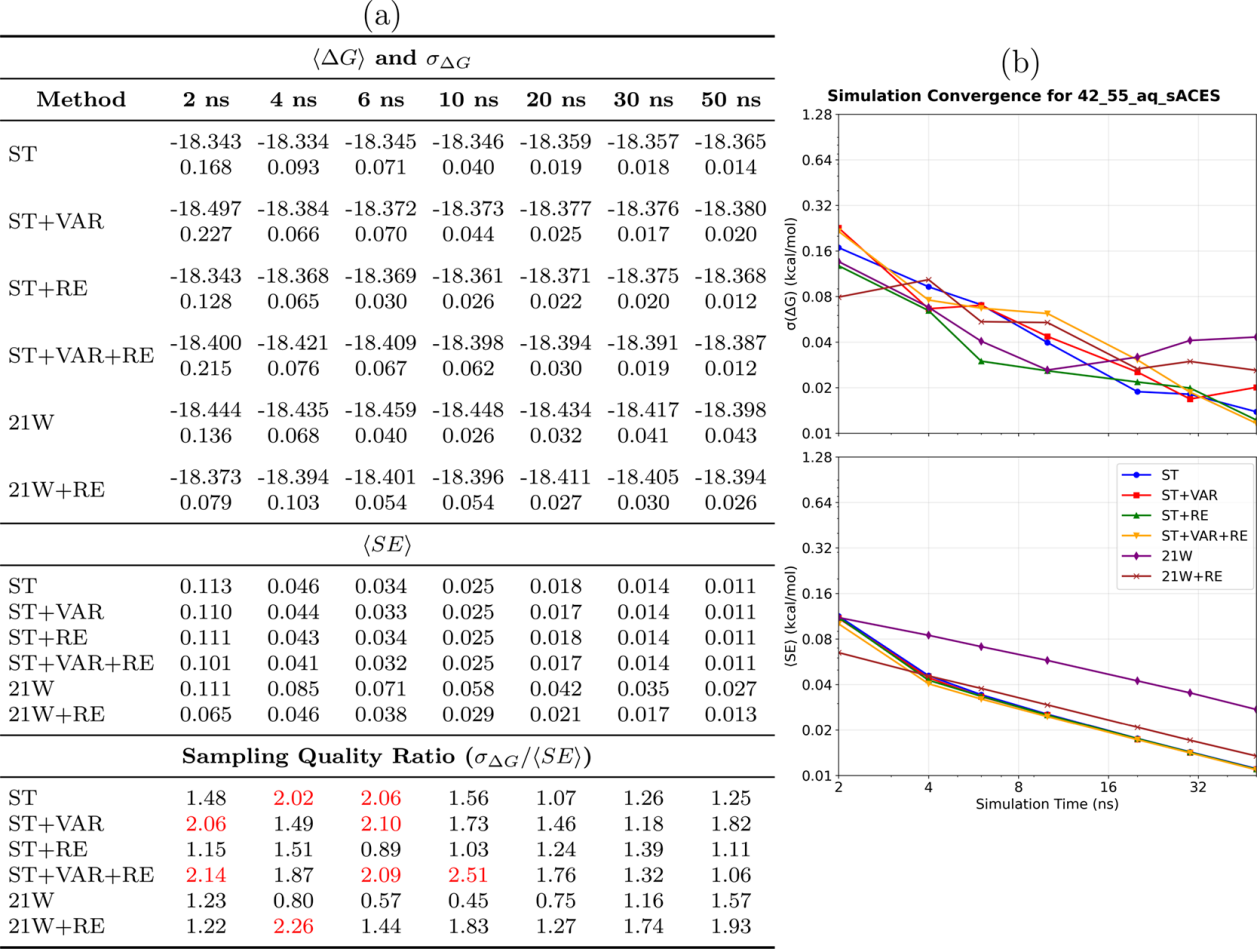
Convergence analysis for the 42→55_aq,sACES_ system (single-torsion ACES). **(a) Performance summary**: Averaged ⟨Δ*G*⟩ and σ_Δ*G*_ (kcal/mol) from 8 simulations (upper section); ⟨SE⟩ (middle section); sampling quality ratio σ_Δ*G*_/⟨SE⟩ (lower section, red if >2.0, bold red if >5.0). Sampling diagnostic: ⟨SE⟩ ≤ σ_Δ*G*_. **(b) Temporal convergence**: σ_Δ*G*_ and ⟨SE⟩ versus time. **Methods** (Table 1): ST, ST+VAR, ST+RE, ST+VAR+RE, 21W, 21W+RE. **sACES observations**: Single-torsion ACES effectively addresses the primary torsional barrier in the 42→55 transformation, achieving substantially improved conformational convergence. Thermodynamic integration with replica exchange markedly diminishes sampling errors, demonstrating a synergistic advantage when conformational barriers are reduced through ACES modifications.

#### 4.4.1 Conformational Sampling Limitations in Standard Methods

In the absence of ACES enhancement, the 42→55 transformation demonstrates inadequate conformational sampling for standard SAMTI variants. The sampling quality diagnostic (⟨*SE*⟩ ≪ σ_Δ*G*_) indicates that simulation replicas explore distinct conformational regions rather than achieving equilibrium sampling.

For the standard 42→55_aq_ and 42→55_com_ systems, basic SAMTI variants (ST, ST+VAR) and conventional methods (21W) exhibit catastrophic sampling deficiencies with quality ratios exceeding 5.0 (bold red in the tables). In contrast, ST+VAR+RE achieves acceptable conformational sampling with ratios near unity (0.92–1.44 at 50 ns), demonstrating that replica exchange effectively addresses the fundamental time scale separation between alchemical and conformational coordinates. However, ACES enhancement dramatically accelerates convergence: ST+VAR+RE requires 30–50 ns to achieve sub-0.02 kcal/mol precision, whereas ST+VAR+RE (sACES) achieves similar precision within 10–20 ns, representing 2–3× speedup. This performance difference arises from kinetic barriers associated with torsional rotation that are reduced through ACES modifications rather than overcome through enhanced sampling alone.

#### 4.4.2 Cooperative Conformational Transitions and Time Scale Analysis

The enhanced performance of mACES relative to sACES underscores the significance of cooperative conformational changes in the 42→55 transformation. A systematic comparison across ACES variants illustrates this progression: sACES systems (Figures 8 and 9) exhibit notable improvements over standard methods, whereas mACES systems (Figures 10 and 11) achieve even more pronounced convergence enhancements. While sACES addresses the primary torsional barrier, mACES facilitates the coordinated rotation of multiple dihedral angles, enabling a more comprehensive exploration of conformationally relevant states.

**Figure 9:**
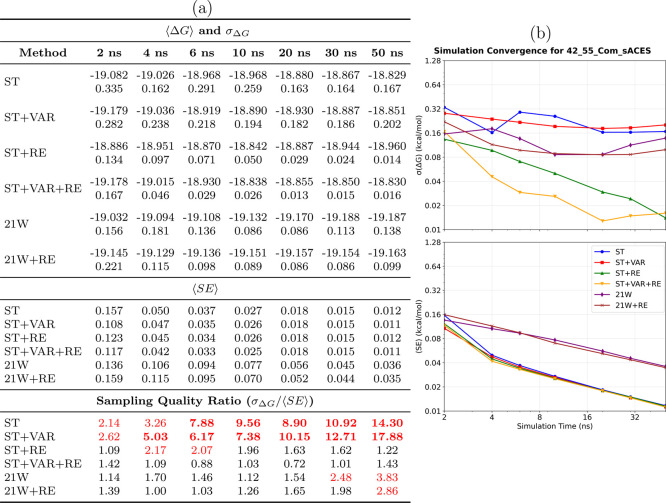
Convergence analysis for the 42→55_com,sACES_ system (single-torsion ACES, protein-bound). **(a) Performance summary**: Averaged ⟨Δ*G*⟩ and σ_Δ*G*_ (kcal/mol) from 8 simulations (upper section); ⟨SE⟩ (middle section); sampling quality ratio σ_Δ*G*_/⟨SE⟩ (lower section, red if >2.0, bold red if >5.0). Sampling diagnostic: ⟨SE⟩ ≤ σ_Δ*G*_. **(b) Temporal convergence**: σ_Δ*G*_ and ⟨SE⟩ versus time. **Methods** (Table 1): ST, ST+VAR, ST+RE, ST+VAR+RE, 21W, 21W+RE. **sACES+protein observations**: SAMTI components with sACES address both alchemical and conformational sampling challenges in complex biomolecular environments. Protein binding site complexity reduces replica exchange efficacy compared to an aqueous environment, underscoring the necessity of considering system complexity when assessing enhanced sampling strategies.

**Figure 10:**
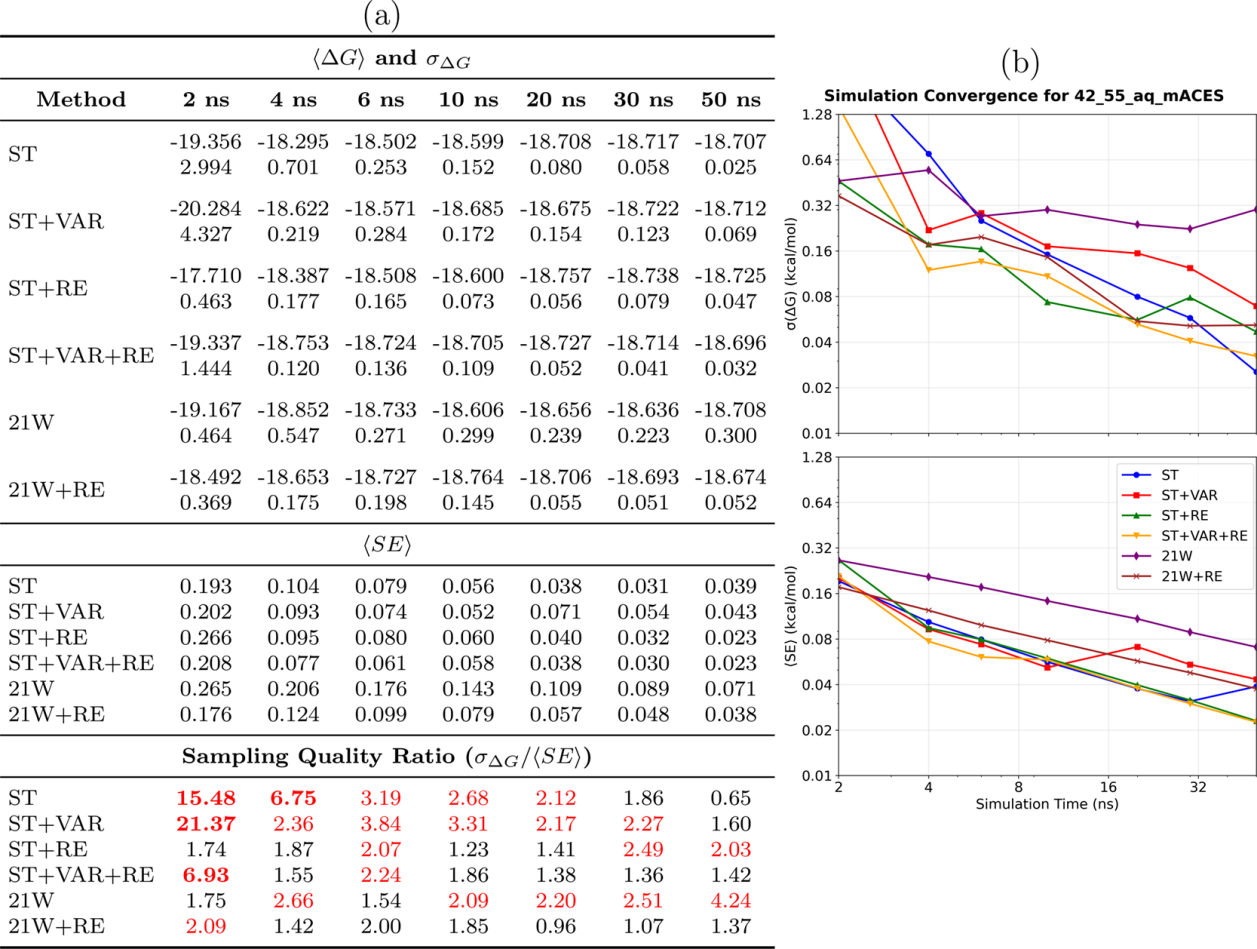
Convergence analysis for the 42→55_aq,mACES_ system (multiple-torsion ACES). **(a) Performance summary**: Averaged ⟨Δ*G*⟩ and σ_Δ*G*_ (kcal/mol) from 8 simulations (upper section); ⟨SE⟩ (middle section); sampling quality ratio σ_Δ*G*_/⟨SE⟩ (lower section, red if >2.0, bold red if >5.0). Sampling diagnostic: ⟨SE⟩ ≤ σ_Δ*G*_. **(b) Temporal convergence**: σ_Δ*G*_ and ⟨SE⟩ versus time. **Methods** (Table 1): ST, ST+VAR, ST+RE, ST+VAR+RE, 21W, 21W+RE. **mACES observations**: Multiple-torsion ACES facilitates coordinated rotation of multiple dihedral angles, enabling a comprehensive exploration of conformationally relevant states. System consistently achieves σ_Δ*G*_ < 0.1 kcal/mol within 10 ns, representing most reliable convergence among all tested methods with 2–3× faster convergence than sACES and 5–10× faster than standard methods.

**Figure 11:**
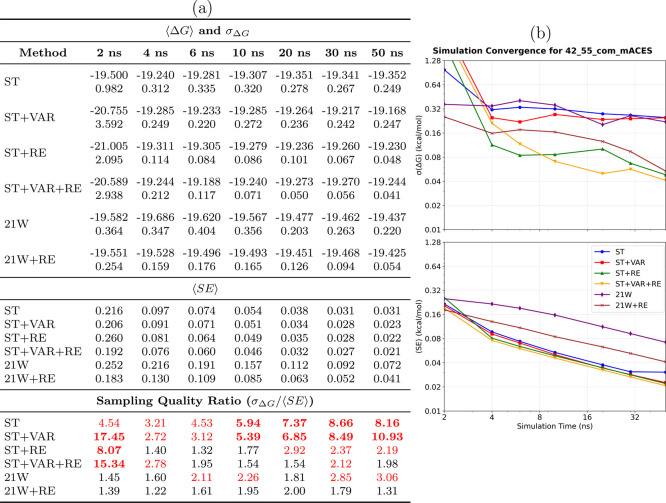
Convergence analysis for the 42→55_com,mACES_ system (multiple-torsion ACES, protein-bound). **(a) Performance summary**: Averaged ⟨Δ*G*⟩ and σ_Δ*G*_ (kcal/mol) from 8 simulations (upper section); ⟨SE⟩ (middle section); sampling quality ratio σ_Δ*G*_/⟨SE⟩ (lower section, red if >2.0, bold red if >5.0). Sampling diagnostic: ⟨SE⟩ ≤ σ_Δ*G*_. **(b) Temporal convergence**: σ_Δ*G*_ and ⟨SE⟩ versus time. **Methods** (Table 1): ST, ST+VAR, ST+RE, ST+VAR+RE, 21W, 21W+RE. **mACES+protein observations**: Most challenging transformation in the test suite, representing the ultimate test of the SAMTI framework. ST+VAR+RE (mACES) provides only a reliable pathway to chemical accuracy (0.041 kcal/mol at 50 ns) in complex biomolecular transformations. Replica exchange provides measurable benefits for conventional methods, but fundamental advantages of the adaptive SAMTI framework are preserved even with enhanced conformational sampling.

Statistical analysis indicates that mACES systems achieve convergence approximately 2–3 times faster than sACES systems and 5–10 times faster than standard methods, attributed to enhanced sampling of cooperative motions crucial for ligand transformation but kinetically hindered in standard simulations. The comparison among standard, sACES, and mACES variants reveals a clear hierarchy of conformational sampling requirements. Standard SAMTI methods excel in addressing sampling challenges along the alchemical coordinate but are inadequate for systems with low conformational degrees of freedom. The 42→55 transformation represents a challenging test case where conformational barriers (characteristic times of ∼10–20 ns) significantly exceed typical alchemical simulation lengths.

### 4.5 Validation of Methodological Unbiasedness

Having established SAMTI’s systematic performance advantages and identified conformational sampling as a key challenge, we now address a fundamental question: do the observed ⟨Δ*G*⟩ differences between SAMTI and conventional TI reflect true methodological bias or simply different convergence rates? Three complementary validation approaches using eight independent simulations per method distinguish these possibilities: (i) temporal convergence analysis tracking whether methods approach the same limiting value at long times; (ii) inter-replicate consistency using the sampling quality ratio σ_Δ*G*_/⟨SE⟩,^83,84^ where values near unity indicate excellent conformational sampling and values exceeding 2.0 reveal severe deficiencies; and (iii) method consensus across the complexity spectrum (see theSupporting Information for complete diagnostic data). Convergence analysis plots with error bars were generated for all eight systems; three representative examples are presented below, with the remaining five systems provided in the Supporting Information (Figures S2–S6).

#### 4.5.1 Simple Systems: Establishing Method Equivalence

The Na^+^ solvation system provides the ideal reference case for validating unbiasedness (Figure 12). At 50 ns, all six methods converge to statistically equivalent values, spanning only 0.025 kcal/mol (75.064–75.089 kcal/mol, with a maximum deviation of 0.024 kcal/mol), which is well within the combined 95% confidence interval of 0.088 kcal/mol. Sampling quality ratios of 0.75–1.73 indicate excellent conformational sampling across all methods. The 7CPI system similarly validates unbiasedness, with all methods achieving ratios of 1.02–1.21 and converging to statistically equivalent values. These results confirm that SAMTI’s adaptive components do not introduce systematic bias; performance differences reflect statistical efficiency gains rather than convergence to incorrect values.

**Figure 12:**
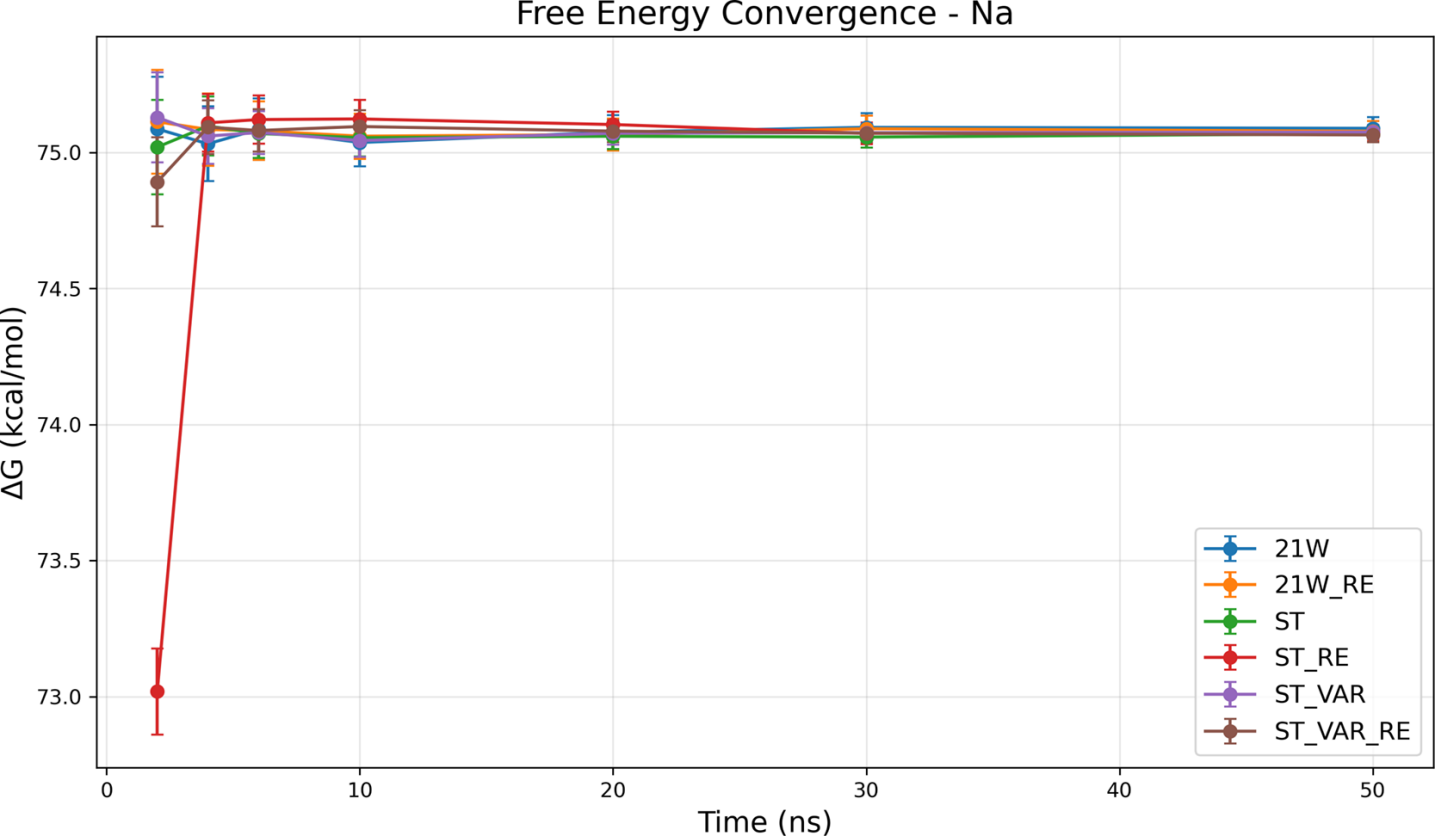
Unbiasedness validation for Na^+^ solvation. All methods converge to statistically equivalent values (75.06–75.09 kcal/mol) with sampling quality ratios of 0.75–1.73, confirming SAMTI introduces no systematic bias. Error bars represent the standard error across eight independent simulations.

#### 4.5.2 Intermediate Complexity: Distinguishing Bias from Convergence

The 42→55_com_ transformation without ACES (Figure 13) reveals the distinction between bias and incomplete convergence. At 50 ns, SAMTI variants (−16.98 to −17.15 kcal/mol) and conventional methods (−15.46 to −15.66 kcal/mol) show a 1.5 kcal/mol offset. However, three observations confirm this reflects convergence rates, not bias: (1) all SAMTI variants converge to mutually consistent values despite different adaptive components; (2) conventional methods drift continuously toward SAMTI values without plateauing; **(3) s**ampling quality ratios (1.33–26.15) reveal severe conformational sampling deficiencies in both SAMTI and conventional methods, with ST+VAR exhibiting catastrophic failure (ratio 26.15). These large ratios confirm observed differences arise from incomplete sampling affecting all methods, not SAMTI-specific bias.

**Figure 13:**
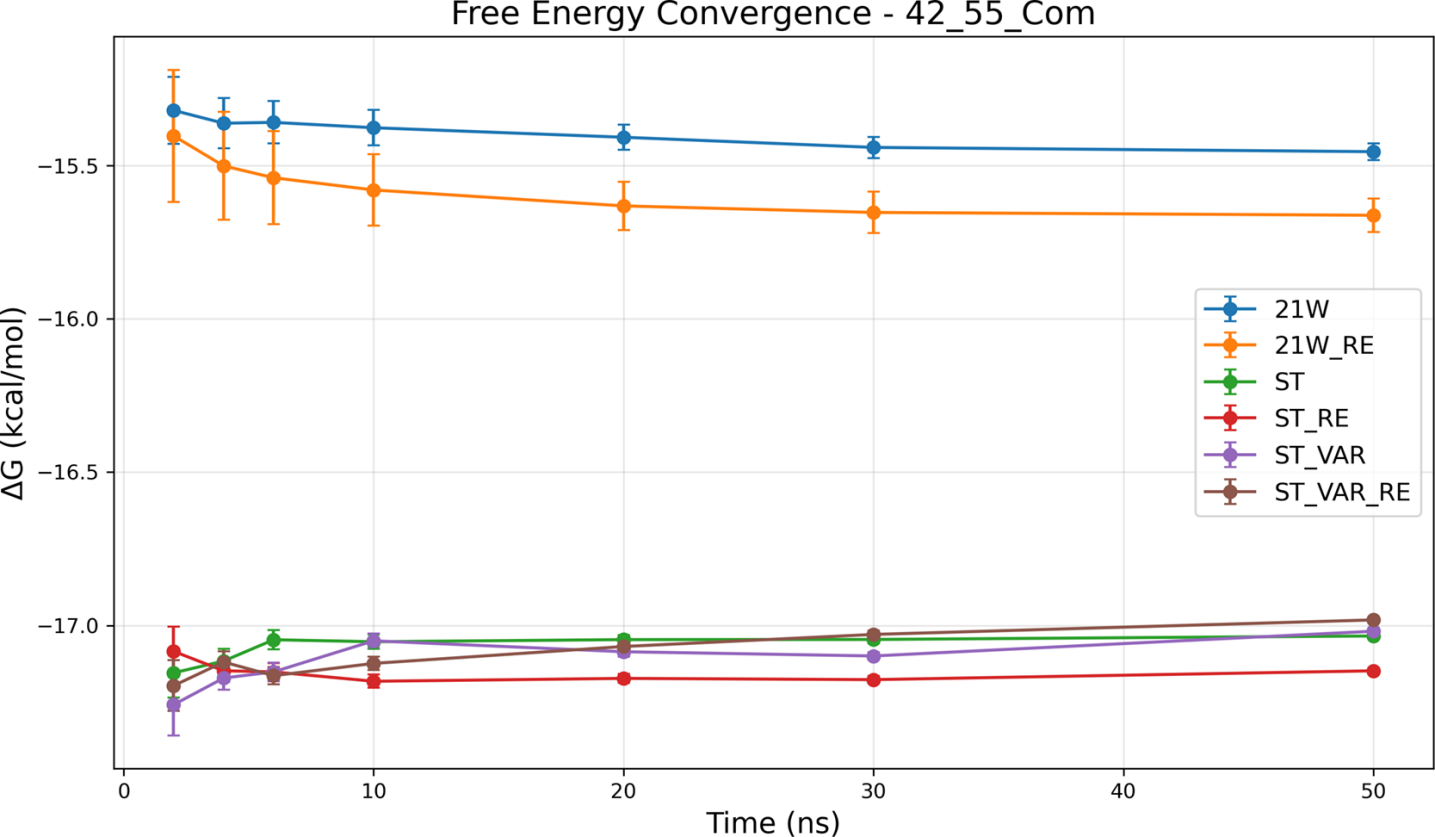
Distinguishing bias from incomplete convergence in 42→55_com_. SAMTI variants show internal consistency (−16.98 to −17.15 kcal/mol), while conventional methods drift continuously (−15.46 to −15.66 kcal/mol). Sampling quality ratios (1.33–26.15) confirm differences arise from incomplete conformational sampling, not systematic bias.

#### 4.5.3 High Complexity: Validation through Enhanced Sampling

The 42→55_com,mACES_ system (Figure 14) provides the most compelling unbiasedness evidence. At 50 ns, conventional methods show poor sampling (ratios 3.06 for 21W, 1.31 for 21W+RE) with large uncertainties and continued drift. SAMTI without ACES exhibits catastrophic failures (ratios 19.09–26.15), where replicates are trapped in distinct conformational basins. Only ST+VAR+RE (mACES) achieves reliable convergence (σ_Δ*G*_ = 0.041 kcal/mol, ratio of 1.98). The systematic improvement with successive SAMTI components—from ratio 26.15 (no ACES) to 1.43 (sACES) to 1.98 (mACES)—without shifts in limiting free energy values among well-sampled methods, demonstrates that enhancements accelerate convergence to the correct thermodynamic value rather than introducing bias.

**Figure 14:**
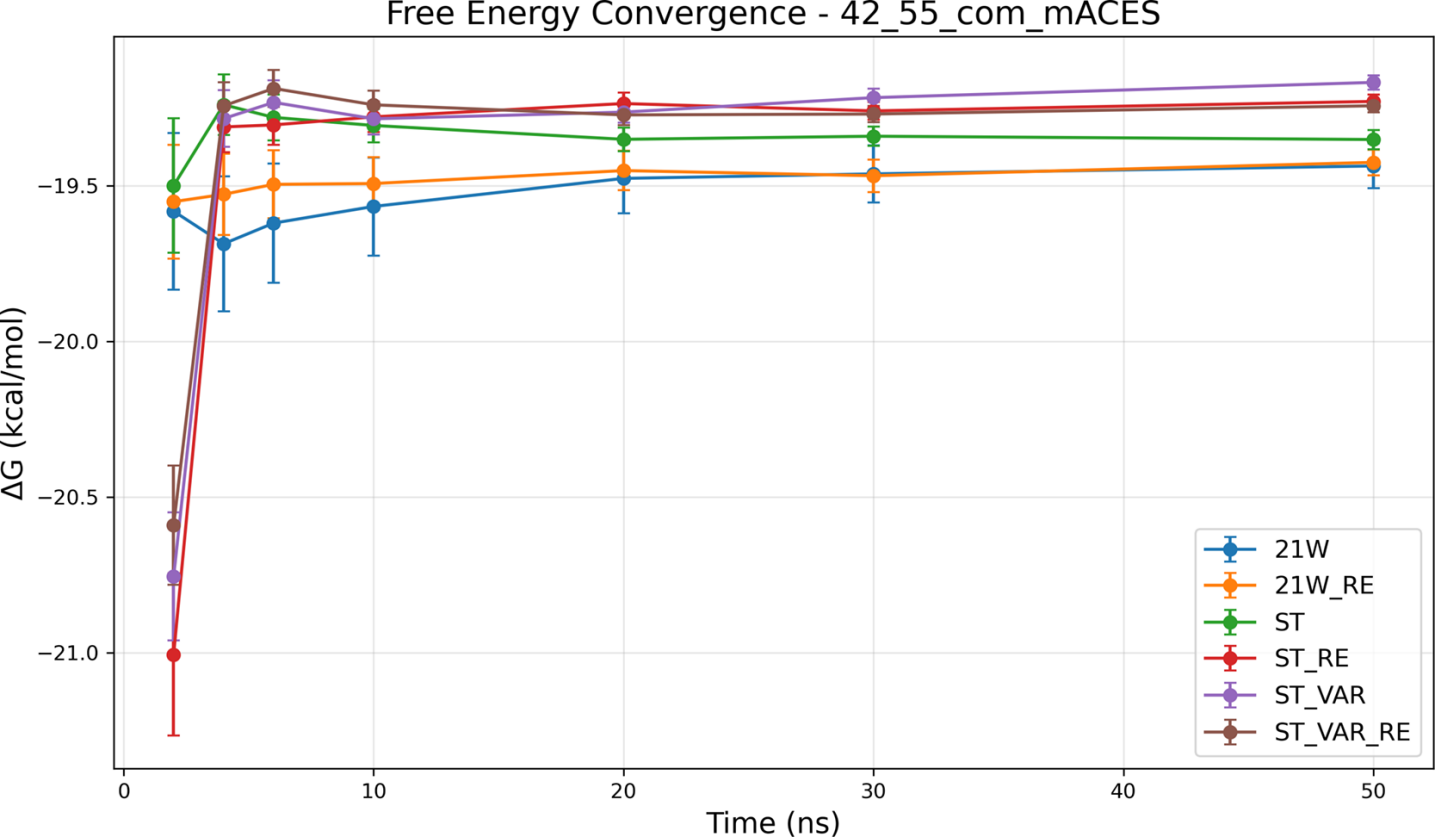
Unbiasedness validation for the most challenging 42→55_com,mACES_ transformation. Only ST+VAR+RE (mACES) achieves adequate sampling (σ_Δ*G*_ = 0.041 kcal/mol, ratio 1.98). Systematic improvement (ratios of 19.09–26.15 without ACES to 1.31–1.98 with mACES) without shifts in limiting values demonstrates SAMTI accelerates convergence without introducing bias.

#### 4.5.4 Summary

Comprehensive analysis across the complexity spectrum confirms SAMTI’s unbiasedness. For well-sampled systems (Na^+^, 7CPI), all methods converge to statistically identical values (maximum deviations of 0.024 and 0.132 kcal/mol, respectively, within combined uncertainties). For complex systems, the sampling quality ratio (σ_Δ*G*_/⟨SE⟩) provides robust convergence diagnostics: ratios below 1.5 indicate reliable estimates, while ratios exceeding 2.0 reveal inadequate sampling. Complete diagnostic data for all 48 combinations (8 systems × 6 methods) are provided in the Supporting Information. The observed ⟨Δ*G*⟩ differences reflect convergence rates, not systematic bias; SAMTI achieves faster, more reliable convergence to correct thermodynamic values.

### 4.6 Computational Analysis

#### 4.6.1 Sampling Efficiency

The sampling quality diagnostic (⟨SE⟩ ≤ σ_Δ*G*_) provides insights into conformational sampling quality, with ratios approaching unity indicating complete exploration and larger ratios revealing sampling deficiencies. We also assessed the sensitivity of the sampling-efficiency analysis to the sampling interval used to estimate the autocorrelations and *N*_eff_. Recomputing *N*_eff_(λ) at 0.2, 0.4, 1.0, and 2.0 ps for Na^+^ and 42→55_com,sACES_ yields consistent profiles and conclusions across frequencies (Supporting Information, Figure S1), confirming that the 0.2 ps baseline used throughout does not inflate *N*_eff_.

##### 4.6.1.1 Autocorrelation Analysis Method

The effective sample size (*N*_eff_) is determined using autocorrelation-based statistical inefficiency analysis. The statistical inefficiency factor *g* is calculated as

$$g=1+2\sum_{k=1}^{k_{\mathrm{cutoff}}}\rho_{k}$$ 15

where ρ_*k*_ represents the autocorrelation function at lag *k*. The autocorrelation function measures the correlation between the time series of ∂*U*/∂λ and a lagged version of itself. For a time series *X*_*t*_, the autocorrelation at lag *k* is given by

$$\rho_{k}=\frac{\mathrm{Cov}\left(X_{t},X_{t+k}\right)}{\mathrm{Var}\left(X_{t}\right)}$$ 16

The summation in the calculation of *g* is truncated at a cutoff *k*_cutoff_ where the autocorrelation function has decayed to zero. The effective sample size is then computed as *N*_eff_ = *N*/*g*, where *N* is the total number of samples. The sampling efficiency η is defined as the ratio η = *N*_eff_/*N* = 1/*g*, with values approaching unity indicating optimal sampling independence. This approach provides a robust assessment of sampling independence by accounting for temporal correlations in the ∂*U*/∂λ time series. A higher sampling efficiency indicates that the samples are less correlated and therefore provide more information about the underlying distribution.

##### 4.6.1.2 Effective Sample Size and Statistical Inefficiency Results

The analysis of effective sample sizes (*N*_eff_), determined by using this autocorrelation-based statistical inefficiency method, reveals fundamental differences in sampling quality between SAMTI and conventional TI methods. A comprehensive comparison across all eight molecular systems (Figure 15) demonstrates systematic efficiency advantages for ST-based approaches.

**Figure 15:**
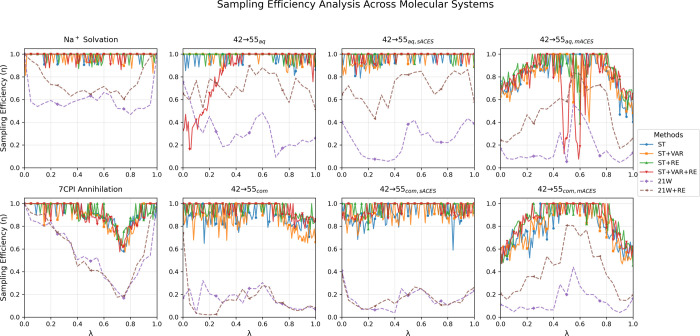
Sampling efficiency analysis across the complete alchemical coordinate. **Layout**: Eight molecular systems arranged in 2 × 4 format. **Top row**: Na^+^ solvation, 42→55_aq_, 42→55_aq,sACES_, and 42→55_aq,mACES_ systems. **Bottom row**: 7CPI annihilation, 42→55_com_, 42→55_com,sACES_, and 42→55_com,mACES_ systems. **Each panel**: Sampling efficiency (η = *N*_eff_/*N*) vs λ, with values approaching 1.0 indicating optimal sampling independence. **Method comparison**: ST-based methods (solid lines) consistently achieve η ≈ 1.0, while conventional TI methods (dashed lines) show systematically lower values. The progression from simple to complex systems demonstrates increasing performance divergence, with ST methods maintaining superior sampling efficiency through the flat potential energy surface achieved by bias potentials. The apparent dips in efficiency for ST+VAR+RE in some regions are an expected consequence of the VAR component, which prioritizes the sampling of high-variance regions to reduce the overall free energy uncertainty.

##### 4.6.1.3 Subpicosecond Sampling Requirements

The observation of (η ≈ 1.0) across most λ points indicates that the autocorrelation time of ∂*U*/∂λ fluctuations often approaches or exceeds this subpicosecond time scale. This finding challenges the conventional understanding of 1–5 ps sampling frequency and suggests that the energy fluctuations driving free energy convergence exhibit significant correlation structures at much shorter time scales than previously recognized.

##### 4.6.1.4 Method-Specific Sampling Performance

ST-based methods exhibit superior sampling efficiency, with mean *N*_eff_ values ranging from 0.91 to 0.94 across all λ points, in contrast to the 0.30–0.50 range observed in conventional TI methods. This significant disparity is directly associated with the ⟨SE⟩ ≤ σ_Δ*G*_ relationship: systems with high *N*_eff_ values demonstrate ⟨SE⟩ ≈ σ_Δ*G*_, indicating comprehensive conformational sampling, whereas systems with low *N*_eff_ values show ⟨SE⟩ ≪ σ_Δ*G*_, indicating incomplete sampling.

##### 4.6.1.5 Origin of ST Efficiency

The enhanced sampling efficiency of ST methods is attributed to the bias potential, which flattens the effective potential energy surface along the alchemical coordinate. This flattening reduces energetic barriers between different λ states, resulting in higher acceptance rates for Monte Carlo moves along the λ axis and improved sampling of subpicosecond dynamics. In contrast, conventional TI methods, which lack this bias potential, exhibit lower acceptance rates and necessitate finer temporal resolution to achieve an equivalent correlation capture.

It is important to note that for the ST+VAR+RE method, the sampling efficiency may appear lower in certain low-variance λ regions. This is an expected and intended consequence of the VAR component, which adaptively allocates more computational effort to high-variance regions. While this may lead to a localized decrease in sampling efficiency in some windows, it results in a more significant reduction in the overall uncertainty of the calculated free energy, which is the primary goal of the SAMTI framework.

##### 4.6.1.6 Replica Exchange Effectiveness

The effectiveness of replica exchange is highly dependent on the system complexity and acceptance rates. For simple systems, such as Na^+^ solvation, the benefits of RE are modest, whereas more complex protein–ligand systems show more substantial improvements (42→55_com_: 0.89–0.95).

##### 4.6.1.7 Connection to Convergence Quality

The correlation between high *N*_eff_ and rapid convergence is evident across all systems. Methods achieving *N*_eff_ > 0.9 consistently demonstrate ⟨SE⟩ ≈ σ_Δ*G*_ relationships and superior convergence properties. Conversely, methods with *N*_eff_ < 0.5 exhibit ⟨SE⟩ ≪ σ_Δ*G*_ and require extended simulation times to achieve comparable accuracy.

#### 4.6.2 Computational Cost

Computational cost assessment for free energy calculations must account for both the per-replica efficiency and the number of parallel replicas required. Table 2 presents measured performance (nanoseconds/day) from representative SAMTI and conventional TI runs for two systems spanning the complexity spectrum: Na^+^ solvation and the protein–ligand transformation 42→55_com,sACES_. For each method configuration, we report (1) per-replica average ns/day derived from AMBER’s total wall-time metric, (2) total parallel throughput (raw) accounting for concurrent replica execution (8 replicas for SAMTI methods; 21 windows for TI methods), and (3) replica-exchange acceptance probability for RE-enabled methods. Rows marked with an asterisk in Table 2 denote SAMTI 42→55_com,sACES_ runs performed on 4-GPU A100 nodes; all other runs used 8-GPU RTX 3090 nodes.

**Table 2:** Performance Comparison (ns·day^–1^)­^a^

system	method	Avg (ns day^–1^)	Thru (raw)	RE acc.
Na	ST	370.53	2964.2	n/a
Na	ST+VAR	376.62	3013.0	n/a
Na	ST+RE	310.26	2482.1	0.130
Na	ST+VAR+RE	310.06	2480.5	0.114
Na	21W	154.35	3241.4	n/a
Na	21W+RE	105.56	2216.9	0.503
42→55_com,sACES_^*^	ST	67.04	536.3	n/a
42→55_com,sACES_^*^	ST+VAR	64.92	519.3	n/a
42→55_com,sACES_^*^	ST+RE	65.84	526.7	0.185
42→55_com,sACES_^*^	ST+VAR+RE	82.68	661.4	0.140
42→55_com,sACES_	21W	56.23	1180.8	n/a
42→55_com,sACES_	21W+RE	44.76	940.1	0.422

a For each configuration, we report per-replica average nanosecond day^–1^ (from AMBER total wall-time), total parallel throughput (raw), and replica-exchange acceptance probability for RE-enabled runs. SAMTI runs use 8 concurrent replicas; TI runs use 21. Rows marked with an asterisk indicate SAMTI 42→55_com,sACES_ runs performed on 4-GPU A100 nodes; all other runs used 8-GPU RTX 3090 nodes.

##### 4.6.2.1 AMBER Reporting Limitations

AMBER does not provide detailed timing breakdowns for individual computational stages (prescan, bias construction, replica exchange bookkeeping, logging, production MD). Instead, the software reports only total wall time, from which the average nanosecond/day performance metric is calculated. For the 50 ns simulations analyzed here, setup overhead (equilibration, initial energy minimization) requires only a few seconds and is negligible compared to the multihour production runs. Furthermore, itemized per-stage timing is not meaningfully separable because multiple stages execute concurrently: GPU kernels handle force evaluation and integration, while CPU threads manage replica exchange proposals, bias updates, and I/O operations. Any attempt to partition the wall time into sequential components would therefore misrepresent the actual parallel execution model.

##### 4.6.2.2 Performance Variability and Load Balancing

All measurements were obtained on a campus shared computing cluster where multiple users’ jobs compete for node resources. Absolute throughput values are therefore subject to background load fluctuations and queue placement variability, making precise cost comparisons difficult. Most calculations utilized 8-GPU RTX 3090 nodes; the SAMTI 42→55_com,sACES_ runs were performed on 4-GPU A100 nodes (marked with * in Table 2). For conventional 21-window TI methods, GPU counts that do not divide evenly by 21 create load imbalance, leading to underutilization and suboptimal aggregate throughput that does not scale linearly with the window count. SAMTI methods with 8 replicas achieve better load distribution on common 4- or 8-GPU configurations.

##### 4.6.2.3 Accuracy versus Cost

While per-replica nanoseconds per day provides a direct performance metric, translating this into an “accuracy-versus-cost” curve requires quantifying accuracy gains, which can only be assessed qualitatively in this context. As demonstrated throughout the Results section, SAMTI methods achieve substantially lower statistical uncertainty (σ_Δ*G*_) than conventional approaches at equivalent simulation lengths. However, the magnitude of improvement varies by system complexity, transformation type, and convergence regime (early-stage rapid improvement vs late-stage asymptotic behavior). Rather than prescribing a single accuracy-cost relationship, we present the measured nanoseconds/day and parallel throughput as practical indicators, allowing readers to evaluate trade-offs based on their specific accuracy requirements and available computational resources.

#### 4.6.3 Performance Summary

Table 3 presents a comprehensive three-way comparison of SAMTI’s optimal method (ST+VAR+RE), enhanced conventional TI with high-frequency replica exchange (21W+RE), and standard conventional TI (21W) across all eight molecular systems at both intermediate (10 ns) and final (50 ns) simulation durations. The systematic analysis identifies several key patterns: (1) **Replica exchange effectiveness**: High-frequency replica exchange (21W+RE) provides significant improvements over standard TI (21W), demonstrating the value of enhanced conformational sampling in conventional methods; (2) **SAMTI superiority**: SAMTI (ST+VAR+RE) systematically outperforms both conventional approaches, with particularly notable advantages in complex transformations; (3) **Rapid convergence**: SAMTI methods frequently achieve at 10 ns what conventional methods require 50 ns to accomplish; and (4) **Enhanced reliability**: SAMTI consistently maintains its performance even in challenging protein-bound environments where conventional methods fail.

**Table 3:** Comprehensive Performance Comparison between SAMTI (ST+VAR+RE), Enhanced Conventional Methods (21W+RE), and Standard Conventional TI (21W) across All Eight Molecular Systems^a^

system	method	⟨Δ*G*⟩ (10 ns)	σ_Δ*G*_ (10 ns)	⟨Δ*G*⟩ (50 ns)	σ_Δ*G*_ (50 ns)
Na^+^ Solvation	21W	75.037	0.080	75.089	0.031
	21W+RE	75.060	0.086	75.078	0.038
	ST+VAR+RE	75.096	0.053	75.064	0.031
7CPI Annihilation	21W	12.292	0.212	12.611	0.067
	21W+RE	12.312	0.267	12.645	0.079
	ST+VAR+RE	12.722	0.095	12.707	0.040
42→55_aq_	21W	–15.394	0.145	–15.373	0.163
	21W+RE	–15.297	0.024	–15.318	0.056
	ST+VAR+RE	–15.249	0.058	–16.552	0.011
42→55_com_	21W	–15.377	0.134	–15.455	0.061
	21W+RE	–15.580	0.164	–15.663	0.072
	ST+VAR+RE	–17.124	0.028	–16.982	0.013
42→55_aq,sACES_	21W	–18.448	0.026	–18.398	0.043
	21W+RE	–18.396	0.054	–18.394	0.026
	ST+VAR+RE	–18.398	0.062	–18.387	0.012
42→55_com,sACES_	21W	–19.132	0.086	–19.187	0.138
	21W+RE	–19.151	0.089	–19.163	0.099
	ST+VAR+RE	–18.838	0.026	–18.830	0.016
42→55_aq,mACES_	21W	–18.606	0.299	–18.708	0.300
	21W+RE	–18.764	0.145	–18.674	0.052
	ST+VAR+RE	–18.705	0.109	–18.696	0.032
42→55_com,mACES_	21W	–19.567	0.356	–19.437	0.220
	21W+RE	–19.493	0.165	–19.425	0.054
	ST+VAR+RE	–19.240	0.071	–19.244	0.041

a Values show averaged free energy differences ⟨Δ*G*⟩ (kcal/mol) and standard deviations σ_Δ*G*_ (kcal/mol) at intermediate (10 ns) and final (50 ns) simulation times. Key observations: (1) Replica exchange (21W+RE) provides significant improvements over standard TI (21W), demonstrating 1.5-2× reductions in σ_Δ*G*_; (2) SAMTI (ST+VAR+RE) systematically outperforms both conventional methods with 2-5× additional reductions in σ_Δ*G*_ across complex systems; (3) Early convergence advantages where SAMTI’s 10 ns performance often exceeds conventional 50 ns results; (4) Most dramatic improvements in protein-bound systems where conventional methods show persistent large uncertainties. The progression from simple (Na^+^) to complex (42→55_com,mACES_) systems reveals increasing SAMTI advantages, while validating the effectiveness of high-frequency replica exchange as an intermediate enhancement.

### 4.7 Summary: Complete Framework Performance

The systematic performance evaluation demonstrates that SAMTI achieves its design objectives of improved accuracy, faster convergence, and enhanced computational efficiency. The quantitative results establish that each component contributes synergistically to overall performance improvements, with benefits scaling systematically with molecular complexity. The complete ST+VAR+RE (mACES) framework consistently achieves σ_Δ*G*_ < 0.1 kcal/mol within 10 ns for complex transformations. The underlying mechanistic origins of these performance improvements are analyzed in the following section.

## 5 Discussion

The SAMTI framework offers a methodologically integrated approach to free energy calculations, effectively addressing the long-standing limitations of conventional thermodynamic integration through four coordinated components: serial tempering (ST), variance-adaptive resampling (VAR), replica exchange (RE), and alchemical enhanced sampling (ACES). Each component is designed to tackle a specific computational challenge: insufficient phase-space overlap between thermodynamic states, suboptimal resource allocation, conformational sampling limitations, and low conformational degrees of freedom. By simultaneously addressing these interdependent issues, SAMTI provides a systematic strategy for achieving statistically robust and computationally efficient free energy estimates across diverse molecular systems.

### 5.1 Component Contributions to Convergence

The performance of SAMTI is derived from the synergistic interplay of its constituent algorithms, each addressing distinct, yet interconnected, limitations in sampling and estimation. The relative impact of ST, VAR, RE, and ACES varies with system complexity, ranging from simple solvation to complex biomolecular assemblies. This modular adaptability facilitates systematic component selection based on transformation requirements.

#### 5.1.1 Serial Tempering (ST)

ST directly addresses the phase-space overlap problem through fine-grained λ spacing (101 windows vs 21), ensuring high correlation between adjacent states and improved acceptance probabilities. Quantitative improvements vary by system complexity: Na^+^ solvation shows ST achieving 0.051 kcal/mol vs 0.031 kcal/mol for 21W, while 7CPI annihilation demonstrates more substantial gains (ST: 0.040 kcal/mol vs 21W: 0.067 kcal/mol), reflecting ST particular effectiveness for systems with complex variance profiles.

#### 5.1.2 Variance-Adaptive Resampling (VAR)

The VAR component addresses the inefficiency in resource allocation inherent in the uniform sampling approaches. Traditional TI allocates equal computational effort to all λ windows, irrespective of their statistical uncertainty, resulting in oversampling of low-variance regions and undersampling of high-variance regions. VAR implements the Neyman optimal allocation by continuously monitoring the variance of $\frac{\partial U}{\partial \lambda }$ at each window and dynamically adjusting sampling probabilities proportionally:

$$\frac{t_{\mathrm{sampling}}\left(\lambda \right)}{t_{\mathrm{total}}}\propto \sigma^{2}\left(\lambda \right)$$

This mechanism ensures that computational resources are directed where they provide the greatest reduction in the overall integration error. This approach is particularly effective for systems exhibiting heterogeneous variance distributions along the λ-pathway, such as those involving changes in net charge. The quantitative impact is demonstrated in Figure 7: for the Na^+^ system, VAR concentrates sampling in the high-variance middle region (λ ≈ 0.3 – 0.6), achieving a 4.2× concentration ratio compared to uniform sampling, while for the 42→55_aq,sACES_ system, VAR redistributes sampling toward regions of statistical uncertainty, demonstrating adaptive resource allocation for conformational challenges. This performance enhancement stems from the VAR ability to automatically detect high-variance λ windows and proportionally allocate computational effort according to Neyman optimal allocation principles, achieving optimal resource allocation through adaptive sampling density modulation. Complementary network-design approaches, such as DiffNet, optimize pairwise measurement graphs across congeneric series to minimize total uncertainty under fixed computational budgets.^85^

#### 5.1.3 Replica Exchange (RE)

The Replica Exchange (RE) methodology addresses the challenges associated with conformational sampling limitations that arise when complex biomolecular systems become trapped in local energy minima. Despite optimal λ spacing (ST) and resource allocation (VAR), a single simulation trajectory may not adequately explore all pertinent conformational states within feasible simulation durations. RE mitigates this issue by executing multiple independent simulations concurrently and periodically attempting to exchange configurations between replicas at varying λ values. This approach enables conformations that are energetically favorable at one λ state to be transferred to other λ values, facilitating the overcoming of local barriers and thereby enhancing the conformational sampling efficiency of the entire ensemble. This is particularly critical for protein–ligand systems, where binding site flexibility results in multiple minima that must be sampled for accurate free energy estimation. The quantitative benefits of RE are system-dependent: simple systems such as Na^+^ solvation exhibit modest improvements (ST+VAR+RE: 0.031 kcal/mol vs ST+VAR: 0.045 kcal/mol), whereas complex protein–ligand systems demonstrate significant gains. For the 42→55_com_ system, RE enables convergence where ST+VAR fails, and in the challenging 42→55_com,mACES_ system, only ST+VAR+RE achieves reliable convergence (0.041 kcal/mol at 50 ns). Thus, the RE is indispensable for systems with substantial conformational complexity.

### 5.2 Microscopic Sampling Efficiency and Time Scale Separation

The fundamental relationship of ⟨SE⟩ ≤ σ_Δ*G*_ serves as a robust diagnostic tool for evaluating the completeness of conformational sampling across alchemical states. Our analysis indicates that this inequality approaches equality only when all simulation replicas explore an identical conformational space, a condition systematically achieved by ST-based methods but seldom by conventional TI approaches.

#### 5.2.1 Statistical Significance

Conducting eight independent simulations per method facilitates a robust statistical assessment. Performance differences between SAMTI variants and conventional methods achieve statistical significance within 95% confidence intervals. In the 7CPI system, ST+VAR achieves 0.036 ± 0.013 kcal/mol compared to 0.067 ± 0.024 kcal/mol for 21W (*p* < 0.05). Systematic improvements across all test systems underscore the general effectiveness of SAMTI.

#### 5.2.2 Enhanced Sampling Efficiency

Autocorrelation analysis (detailed in Results section) reveals that ST methods achieve a sampling efficiency of η = *N*_eff_/*N* ≈ 1.0 at frequencies of 0.2 ps, thereby challenging traditional sampling protocols of 1–5 ps. This near-unity efficiency suggests a rapid decay of autocorrelation and minimal statistical inefficiency.

The bias potential significantly modifies the correlation structure of ∂*U*/∂λ, facilitating near-independent subpicosecond sampling. In contrast, conventional TI demonstrates lower efficiency (η = 0.30 – 0.50, *g* ≈ 2 – 3), particularly in protein–ligand systems where efficiency is even lower (η < 0.2, *g* > 5). This reduced efficiency is evidenced by ⟨SE⟩ ≪ σ_Δ*G*_, indicating incomplete conformational sampling across the replicas.

#### 5.2.3 System-Dependent Sampling Diagnostics and Method Reliability

The correlation between ⟨SE⟩ and σ_Δ*G*_ provides critical insights into the reliability of methods across different system complexities. The Na^+^ system exemplifies the robustness of this diagnostic, achieving ⟨SE⟩ ≈ σ_Δ*G*_ across all methods, thereby confirming adequate sampling for simple electrostatic transformations. Conversely, the 42→55 systems without ACES reveal fundamental limitations: significant deviations between ⟨SE⟩ and σ_Δ*G*_ indicate severe sampling deficiencies, while notable differences between SAMTI and TI results suggest that neither approach is reliable without enhanced conformational sampling. This diagnostic relationship thus serves as an essential quality control metric, facilitating a real-time assessment of whether free energy calculations can be trusted or require methodological enhancement.

#### 5.2.4 Physical Basis

The bias potential of ST flattens the effective energy surface along the alchemical coordinate, thereby reducing conformational barriers and resulting in (1) higher transition acceptance rates, (2) reduced correlation times, and (3) enhanced conformational exploration. This modified landscape enables ST to sample reduced barriers, whereas conventional TI encounters full energetic barriers, thereby explaining the pronounced N_eff_ advantage in complex systems.

#### 5.2.5 Protocol Design Implications

These findings challenge the conventional sparse sampling (1–5 ps), which may underestimate the correlation structure and inflate convergence estimates. Achieving η ≈ 1.0 necessitates subpicosecond sampling, suggesting that protocols should prioritize frequent data collection over extended duration. Real-time computation of *g* provides quality diagnostics: *g* < 2 indicates adequate resolution, while *g* > 5 signals the need for methodological improvements. The correlation between high *N*_eff_ and ⟨SE⟩ ≈ σ_Δ*G*_ values enables adaptive protocol adjustment.

### 5.3 ACES Integration and Extended SAMTI Framework

The integration of Alchemical Enhanced Sampling (ACES) with SAMTI addresses a fundamental limitation identified in the 42→55 transformation studies: the inability of conventional enhanced sampling methods to overcome conformational barriers with characteristic time scales exceeding simulation lengths. The extended ST+VAR+RE with the ACES framework offers a comprehensive solution to multidimensional sampling challenges in complex alchemical transformations.

#### 5.3.1 ACES as the Fourth Essential Component

ACES emerges as a crucial fourth component of the SAMTI framework, specifically addressing conformational sampling limitations that cannot be resolved through the alchemical space enhancement alone. While ST, VAR, and RE optimize sampling along the λ coordinate and through replica coordination, ACES creates enhanced sampling pathways for slow conformational degrees of freedom that represent kinetic bottlenecks.

The integration of ACES demonstrates synergistic advantages that scale with molecular complexity. In aqueous systems, ST+VAR+RE (sACES) achieves 0.012 kcal/mol uncertainty for 42→55_aq,sACES_ compared to 0.020 kcal/mol for ST+VAR alone, while mACES further improves the performance (0.032 kcal/mol for 42→55_aq,mACES_). In protein environments, synergistic benefits are more pronounced: ST+VAR+RE (mACES) achieves 0.041 kcal/mol for 42→55_com,mACES_, where standard methods fail, demonstrating the four-component framework’s ability to address interdependent sampling limitations.

#### 5.3.2 Conformational vs Alchemical Sampling Separation

The 42→55 transformation analysis reveals that conformational and alchemical sampling present distinct but coupled challenges. The ⟨SE⟩ ≪ σ_Δ*G*_ relationship observed in standard SAMTI methods reflects fundamental time scale separation: while SAMTI excels at enhanced sampling along the alchemical coordinate (subpicosecond to picosecond time scales), conformational barriers can persist on nanosecond to microsecond time scales.

ACES bridges this time scale gap by selectively reducing conformational barriers while maintaining thermodynamic consistency. The sACES versus mACES comparison demonstrates that the complexity of required enhancement scales with the cooperative nature of conformational changes: single torsion barriers can be addressed through targeted enhancement, while complex transformations requiring coordinated motion benefit from multiple-torsion approaches.

#### 5.3.3 Environment-Dependent Enhanced Sampling Effectiveness

A systematic evaluation of the effectiveness of replica exchange across various ACES variants reveals significant trends that are dependent on the environment. In aqueous sACES systems, the integration of thermodynamic integration (TI) with replica exchange results in notable improvements in ⟨SE⟩ values, indicating an effective synergy between replica exchange (RE) and scaled torsion potentials. However, this enhancement is considerably diminished in protein-bound sACES environments, where increased complexity reduces the effectiveness of RE. In mACES systems, while replica exchange provides measurable benefits for conventional TI methods in both environments, these improvements consistently fall short of those achieved by SAMTI variants. This pattern suggests that environmental complexity influences the effectiveness of enhanced sampling strategies, with the SAMTI adaptive framework maintaining robust performance across diverse chemical environments.

### 5.4 Grid Design Rationale and Validation

The choice of a near-continuous grid (101 uniformly spaced λ windows) in SAMTI is fundamental to minimizing sampling barriers between adjacent thermodynamic states. This dense grid ensures high phase-space overlap, facilitating efficient Monte Carlo transitions along the alchemical coordinate and enabling the ST component to explore λ space effectively. Reducing the number of grid points or using nonuniform spacing would defeat this design principle and compromise the synergy between ST and VAR: ST requires dense spacing to maintain high acceptance probabilities, while VAR (as demonstrated in Figure 7) adaptively redistributes the sampling effort to high-variance regions *within* the fixed grid structure. The combination of dense uniform spacing (ST) and adaptive resource allocation (VAR) addresses orthogonal challenges—phase-space connectivity and statistical efficiency, respectively.

Grid independence has been validated through a control calculation using a denser 201-window layout for the 7CPI system. As reported in the Supporting Information (Table S1), all free energy estimates at 50 ns remain within one combined standard deviation between 101- and 201-window protocols (maximum |Δ|/σ_comb_ = 0.71 for ST+VAR+RE), confirming that the 101-window grid is sufficient for accurate thermodynamic integration. The consistency between grid densities demonstrates that SAMTI’s adaptive components govern convergence behavior rather than grid refinement, validating the 101-window choice as both scientifically sound and computationally efficient.

### 5.5 Synergy and Practical Recommendations

The ST+VAR+RE with ACES implementation demonstrates performance that surpasses any subset of its components through multidimensional synergy: ST constructs finely resolved thermodynamic pathways; VAR optimally allocates resources; RE enhances space exploration; and ACES addresses conformational barriers. This integration results in a robust methodology that can be applied across the molecular system spectrum.

## 6 Conclusion

The SAMTI framework, through its synergistic integration of serial tempering, variance adaptive resampling, replica exchange, and alchemical enhanced sampling, represents a significant advancement in the field of alchemical free energy calculations. Our extensive benchmarking across a diverse array of molecular systems demonstrates that SAMTI consistently addresses the primary limitations of conventional TI, achieving a substantial reduction in statistical uncertainties while maintaining or enhancing computational efficiency.

The principal finding of this study is that the four-component ST+VAR+RE (mACES) configuration offers a robust and reliable solution for even the most challenging alchemical transformations, consistently attaining chemical accuracy (σ_Δ*G*_ < 0.1 kcal/mol) within practical simulation durations.

By transforming free energy calculations from a specialized and often unreliable tool into a more routine and predictable method, SAMTI holds the potential to significantly expedite discovery processes in drug design, materials science, and other areas of molecular engineering. The modular and automated nature of the framework renders it accessible to a broad spectrum of researchers, and its rigorous statistical foundation offers a new level of confidence in the accuracy of the results.

## Supplementary Material



## Data Availability

The full SAMTI functionality will be incorporated into the next major release of the AMBER simulation package.

## Notes

Comparison of 50 ns free energy estimates; sensitivity of sampling efficiency to sampling frequency; convergence analysis with error bars for the 7CPI annihilation system; convergence analysis with error bars; and summary of convergence analysis findings (PDF)
