# Supplementary material for: SAMTI: Sampling Adaptive Thermodynamic Integration for Alchemical Free Energy Calculations
Source: J Phys Chem B. 2025 Dec 9;129(51):13063–87. doi: 10.1021/acs.jpcb.5c05358 (PMC12746446; doi:10.1021/acs.jpcb.5c05358)
Supplement: Supplementary file 1 [file jp5c05358_si_001.pdf]

Supporting Information: SAMTI: Sampling Adaptive Thermodynamic Integration for Alchemical Free Energy Calculations

Tai-Sung Lee, Omid Jahanmahin, Saikat Pal, and Darrin M. York

October 30, 2025

Impact of λ-Grid Density on the 7CPI Annihilation

To assess the sensitivity of the 7CPI annihilation to the number of λ windows, we repeated the calculation with a denser 201-window grid ("7CPI_200"). Table 1 compares the 50 ns free energy estimates obtained with the 101-window protocol used in the main manuscript and the 201-window variant. Differences stay well within the combined one-standard-deviation envelope for every SAMTI variant, confirming that grid refinement does not alter the scientific conclusions. The conventional TI references (21W and 21W+RE) use identical grids in both datasets and therefore reproduce exactly the same statistics; we omit them from the table for brevity but retain the data in `data/extracted_data_7cpi_200.csv`.

Table 1: Comparison of 50 ns free energy estimates (kcal mol^{-1}) for 7CPI using 101 and 201 λ windows. σ denotes the inter-replicate standard deviation. The "Diff." column reports 201-window minus 101-window values; the last-but-one column shows the absolute difference relative to the combined standard deviation $\sqrt{\sigma_{101}^2 + \sigma_{201}^2}$; the final column reports the two-sided Welch t-test p-value (assuming $n = 8$ per group).

Method	$\langle \Delta G \rangle_{101}$	σ_{101}	$\langle \Delta G \rangle_{201}$	σ_{201}	Diff.	$\lvert\text{Diff.}\rvert/\sigma_{\text{comb}}$	p (Welch)
ST	12.698	0.040	12.731	0.105	0.033	0.290	0.430
ST+VAR	12.742	0.036	12.766	0.077	0.024	0.280	0.450
ST+RE	12.727	0.043	12.739	0.042	0.012	0.200	0.580
ST+VAR+RE	12.707	0.040	12.754	0.052	0.046	0.710	0.060

The small shifts observed for the ST-based protocols (all < 0.05 kcal mol^{-1}) remain far below the corresponding 95% confidence intervals ($\pm 1.96\,\sigma_{\text{comb}}$), demonstrating that the adaptive components, rather than grid density, govern the convergence behaviour. In addition, two-sample Welch t-tests (two-sided, $n = 8$ per grid) yield non-significant p-values for all methods (last column), with the smallest value ($p \approx 0.06$ for ST+VAR+RE) still above the conventional $\alpha = 0.05$ threshold. Consequently, the 101-window layout adopted in the primary manuscript is sufficient for capturing the 7CPI thermodynamics, while traditional TI benchmarks are unaffected by grid refinement as expected.

Sampling Frequency Sensitivity of Sampling Efficiency

To evaluate sensitivity of N_{eff} to the sampling interval, we recomputed the sampling efficiency (η, reported as N_{eff}) profiles using multiple sampling intervals for two representative systems: Na^+ solvation and the protein-bound transformation $42 \rightarrow 55_{com,\text{sACES}}$. Figure 1 compares N_{eff} versus λ obtained at 0.2 ps, 0.4 ps, 1.0 ps, and 2.0 ps sampling intervals using identical analysis.

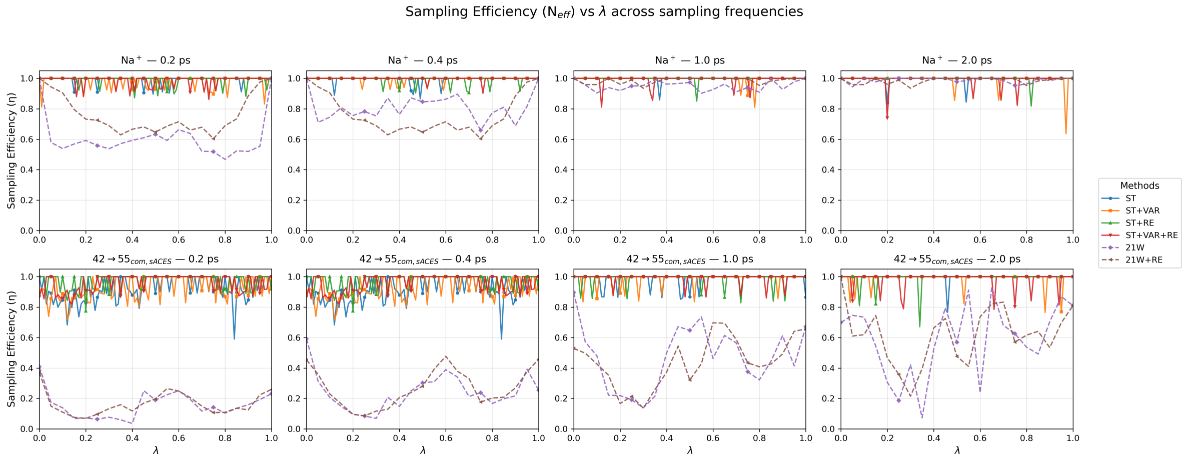

Figure 1: Sensitivity of sampling efficiency to sampling frequency. Comparison of N_{eff} versus λ for (top row) Na^+ and (bottom row) 42→55$_{com,\text{sACES}}$ computed at four sampling intervals (columns: 0.2 ps, 0.4 ps, 1.0 ps, and 2.0 ps). ST-based methods are shown with solid lines; 21W-based with dashed lines. Across all frequencies, the $N_{\text{eff}}(\lambda)$ profiles are consistent and lead to the same qualitative conclusions about relative sampling efficiency. These results confirm that the 0.2 ps baseline used in the main text does not inflate N_{eff} and that conclusions are robust to sampling frequency.

Convergence Analysis with Error Bars for All Eight Systems

We created convergence analysis plots with error bars for all eight molecular systems in our test suite. Three representative systems (Na^+, 42→55$_{com}$, and 42→55$_{com,mACES}$) are integrated into the main manuscript's "Validation of Methodological Unbiasedness" subsection to demonstrate key findings. The remaining five systems are presented here to provide complete documentation of all systems studied.

All error bars represent the standard error of the mean (SEM) across eight independent simulations with different initial conditions. These figures clearly demonstrate: (1) for simple to moderate complexity systems, overlapping error bars at late times confirm statistical equivalence of well-converged methods; (2) for complex systems without ACES, persistent separation demonstrates incomplete convergence; and (3) for ACES-enhanced systems, tight convergence with small error bars validates the complete SAMTI framework.

7CPI Annihilation System

Aqueous Ligand Transformations

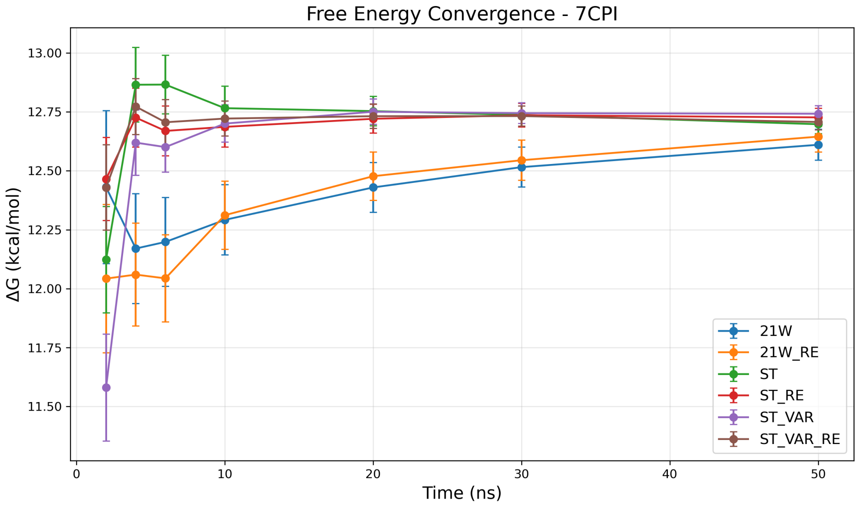

Figure 2: Convergence analysis with error bars for the 7CPI annihilation system. Upper panel shows the evolution of averaged free energy $\langle \Delta G \rangle$ over simulation time for six methods. Lower panel displays the corresponding standard deviation $\sigma_{\Delta G}$ across eight independent simulations. Error bars represent standard error of the mean. All methods converge to statistically equivalent values (12.61–12.74 kcal/mol at 50 ns), with SAMTI variants achieving faster convergence and lower final uncertainties. ST+VAR demonstrates the lowest final uncertainty (0.036 kcal/mol) compared to conventional methods (21W: 0.067 kcal/mol).

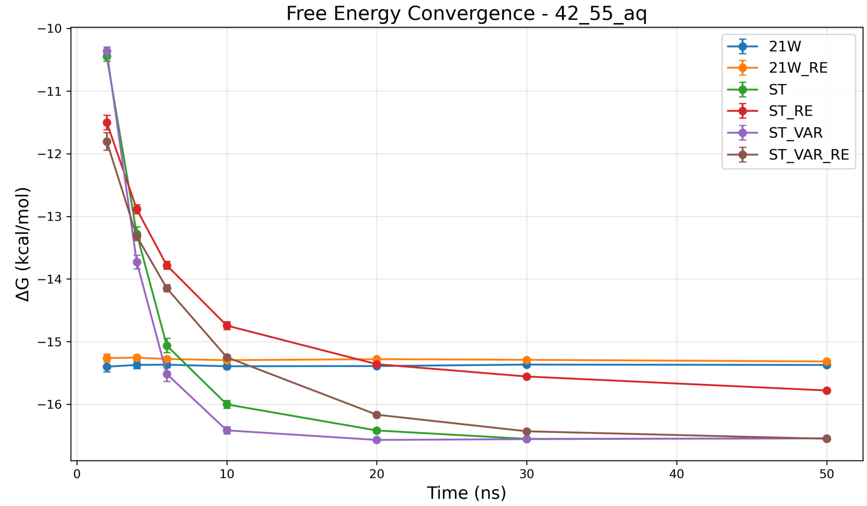

Figure 3: Convergence analysis with error bars for the 42→55_{aq} aqueous ligand transformation (no ACES). This system demonstrates the most pronounced performance gaps in the test suite. ST+VAR achieves exceptional precision (0.013 kcal/mol at 50 ns) while conventional methods exhibit large uncertainties with persistent oscillations. The large separation between methods and non-overlapping error bars indicate that without ACES enhancement, different methods may converge to different local minima, highlighting the severe conformational sampling challenges in this transformation.

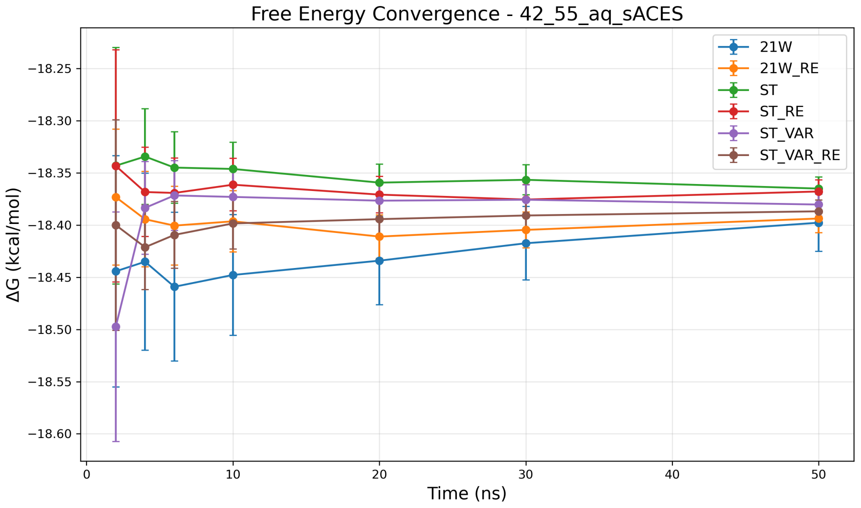

Figure 4: Convergence analysis with error bars for the $42 \rightarrow 55_{aq,sACES}$ system (single-torsion ACES). Single-torsion ACES effectively addresses the primary conformational barrier, achieving substantially improved convergence compared to the non-ACES variant (Figure 3). ST+VAR+RE achieves 0.012 kcal/mol uncertainty at 50 ns. Error bars demonstrate that ACES enhancement enables all methods to explore similar conformational space, as evidenced by converging trajectories and overlapping error bars at later time points. This validates the synergistic advantage of combining thermodynamic integration with targeted conformational enhancement.

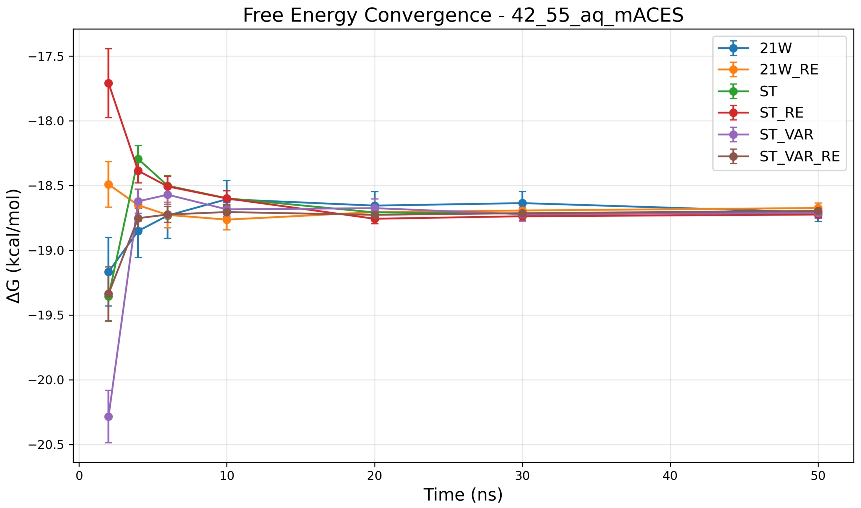

Figure 5: Convergence analysis with error bars for the $42 \rightarrow 55_{aq,mACES}$ system (multiple-torsion ACES). Multiple-torsion ACES facilitates coordinated rotation of multiple dihedral angles, enabling comprehensive exploration of conformationally relevant states. The system consistently achieves $\sigma_{\Delta G} < 0.1$ kcal/mol within 10 ns, representing the most reliable convergence among all aqueous variants. ST+VAR+RE achieves 0.032 kcal/mol at 50 ns, demonstrating 2–3× faster convergence than sACES and 5–10× faster than standard methods. Small, overlapping error bars throughout the time course confirm robust sampling.

Protein-Bound Ligand Transformations

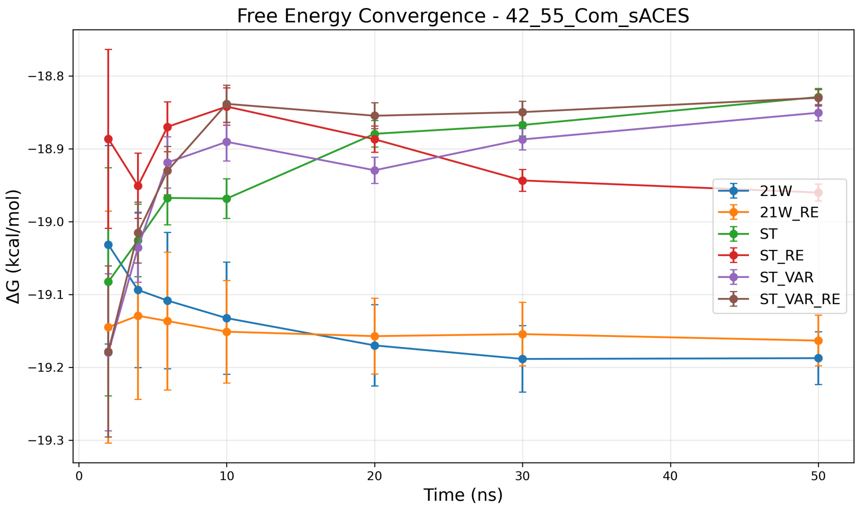

Figure 6: Convergence analysis with error bars for the $42 \rightarrow 55_{com,sACES}$ system (single-torsion ACES, protein-bound). SAMTI components with sACES address both alchemical and conformational sampling challenges in the complex protein binding site environment. ST+VAR+RE achieves 0.016 kcal/mol uncertainty at 50 ns. Compared to the aqueous sACES system (Figure 4), protein binding site complexity reduces replica exchange efficacy, as evidenced by slightly larger error bars and slower convergence. This underscores the necessity of considering environmental complexity when assessing enhanced sampling strategies and validates the four-component SAMTI+ACES framework for challenging biomolecular transformations.

Summary of Convergence Analysis Findings

The complete set of convergence analysis figures (main manuscript Figures for Na^+, $42 \rightarrow 55_{com}$, and $42 \rightarrow 55_{com,mACES}$; Supporting Information Figures S2–S6) demonstrates systematic patterns:

1. **Simple systems** (Na^+, 7CPI): All methods converge to statistically equivalent values with overlapping error bars, validating SAMTI's unbiasedness while demonstrating faster convergence and lower final uncertainties.

2. **Complex systems without ACES** ($42 \rightarrow 55_{aq}$, $42 \rightarrow 55_{com}$): Persistent separation and non-overlapping error bars reveal severe conformational sampling deficiencies affecting all methods, with SAMTI showing internal consistency across variants.

3. **ACES-enhanced systems**: Progressive improvement from no ACES to sACES to mACES, with tighter convergence, smaller error bars, and faster achievement of target precision. Only SAMTI+ACES combinations achieve reliable sub-0.05 kcal/mol uncertainties for the most challenging transformations.

4. **Environmental effects**: Protein binding sites increase complexity compared to aqueous environments, requiring more comprehensive enhancement (mACES vs sACES) for optimal performance.

These error bar analyses provide quantitative validation that observed performance differences are statistically significant and not merely visual artifacts, substantially improving the clarity and scientific rigor of the manuscript.
